# The essential and multifunctional TFIIH complex

**DOI:** 10.1002/pro.3424

**Published:** 2018-04-27

**Authors:** Jenna K. Rimel, Dylan J. Taatjes

**Affiliations:** ^1^ Department of Chemistry & Biochemistry University of Colorado Boulder Colorado 80303

**Keywords:** TFIIH, transcription, Mediator, TFIIE, XPD, XPB, CDK7, THZ1, triptolide, RNA polymerase II pausing

## Abstract

TFIIH is a 10‐subunit complex that regulates RNA polymerase II (pol II) transcription but also serves other important biological roles. Although much remains unknown about TFIIH function in eukaryotic cells, much progress has been made even in just the past few years, due in part to technological advances (e.g. cryoEM and single molecule methods) and the development of chemical inhibitors of TFIIH enzymes. This review focuses on the major cellular roles for TFIIH, with an emphasis on TFIIH function as a regulator of pol II transcription. We describe the structure of TFIIH and its roles in pol II initiation, promoter‐proximal pausing, elongation, and termination. We also discuss cellular roles for TFIIH beyond transcription (e.g. DNA repair, cell cycle regulation) and summarize small molecule inhibitors of TFIIH and diseases associated with defects in TFIIH structure and function.

## Introduction

As expected for a large, multi‐subunit complex, TFIIH serves many biological roles, ranging from DNA repair to transcription to cell cycle regulation. The 10‐subunit TFIIH complex performs these roles primarily, if not exclusively, in the nucleus. However, the dissociable 3‐subunit kinase module (consisting of MAT1, CCNH, and CDK7 in humans) can localize to the cytoplasm. Whereas this review will focus on TFIIH as a regulator of RNA polymerase II (pol II) transcription, we describe some of its other biological roles in later sections. We also note other excellent reviews that focus on TFIIH in transcription and/or DNA repair.^1^, ^2^, ^3^ Given the rapid advances in our understanding of TFIIH structure and function, enabled in part by recent cryoEM data and improved chemical inhibitors, it is timely to summarize these findings in the context of established models of TFIIH function. We start with a description of the composition and structure of TFIIH, followed by sections devoted to (i) transcription regulation and other cellular roles, (ii) pathologies associated with TFIIH function, and (iii) small molecule inhibitors of TFIIH. We conclude with a section that summarizes some outstanding questions that could be addressed in future experiments.

## Composition and Structure of TFIIH

TFIIH is conserved from yeast to humans and its basic composition and function was established through biochemical experiments almost three decades ago.^4^, ^5^, ^6^, ^7^, ^8^, ^9^, ^10^, ^11^ TFIIH is a ten‐subunit complex; seven of these subunits comprise the “core” whereas three comprise the dissociable “CAK” (CDK Activating Kinase) module. Human TFIIH subunits are conserved across eukaryotes (Table 1), with highest conservation among its three catalytic subunits XPB, XPD, and CDK7. The TFIIH core contains XPB, XPD, p62, p52, p44, p34, and p8; the CAK contains MAT1, CCNH, and CDK7.

**Table 1:** Human TFIIH Subunits with Their Gene Names, Yeast Homologs, and Basic Functional Roles are Listed, Along with Their Percent Identity in Bold Versus M. musculus, D. melanogaster, S. pombe, and S. cerevisiae^a^

Human core	Gene	Yeast	Function	*M. musculus*	*D. melanogaster*	*S. pombe*	*S. cerevisiae*
XPB (P19447)	*ERCC3*	Ssl2	3'‐5' ATP‐dependent helicase; translocase	**96%** (P49135)	**69%** (Q02870)	**56%** (O13768)	**55%** (Q00578)
XPD (P18074)	*ERCC2*	Rad3	5'‐3' ATP‐dependent helicase	**98%** (O08811)	**68%** (Q9XYZ2)	**53%** (P26659)	**56%** (P06839)
p62 (P32780)	*GTF2H1*	Tfb1	Structural	**97%** (Q9DBA9)	**45%** (Q96OE8)	**27%** (O13745)	**21%** (P32776)
p52 (Q92759)	*GTF2H4*	Tfb2	Regulates XPB ATPase activity	**99%** (O70422)	**51%** (Q9VURI)	**35%** (P87303)	**45%** (Q02939)
p44 (Q13888)	*GTF2H2*	Ssl1	Regulates XPD ATPase activity	**97%** (Q9JIB4)	**51%** (Q9VNP8)	**40%** (O74995)	**39%** (Q04673)
p34 (Q13889)	*GTF2H3*	Tfb4	Structural	**94%** (Q8VD76)	**56%** (Q8S789)	**31%** (O74366)	**37%** (Q12004)
p8 (Q6XYL4)	*GTF2H5*	Tfb5	Regulates XPB ATPase activity	**99%** (Q8K2X8)	**65%** (B7Z018)	**25%** (Q9HDW3)	**28%** (Q3E7C1)
**CAK**							
MAT1 (P51948)	*MNAT1*	Tfb3	CAK stabilization	**96%** (P51949)	**54%** (Q7KPG8)	**30%** (O94684)	**39%** (Q03290)
Cyclin H (P51946)	*CCNH*	Ccl1	Regulates Kinase Activity	**95%** (Q61458)	**48%** (O76513)	**26%** (P36613)	**36%** (P37366)
CDK7 (P50613)	*CDK7*	Kin28	Kinase	**95%** (Q03147)	**66%** (Q24216)	**48%** (Q12126)	**53%** (P06242)

a Uniprot accession numbers are shown in parenthesis. All percent identities were determined from the protein BLAST tool on the National Center for Biotechnology Information website.

### TFIIH core subunits: XPB, XPD, p62, p52, p44, p34, p8

**Figure 1:**
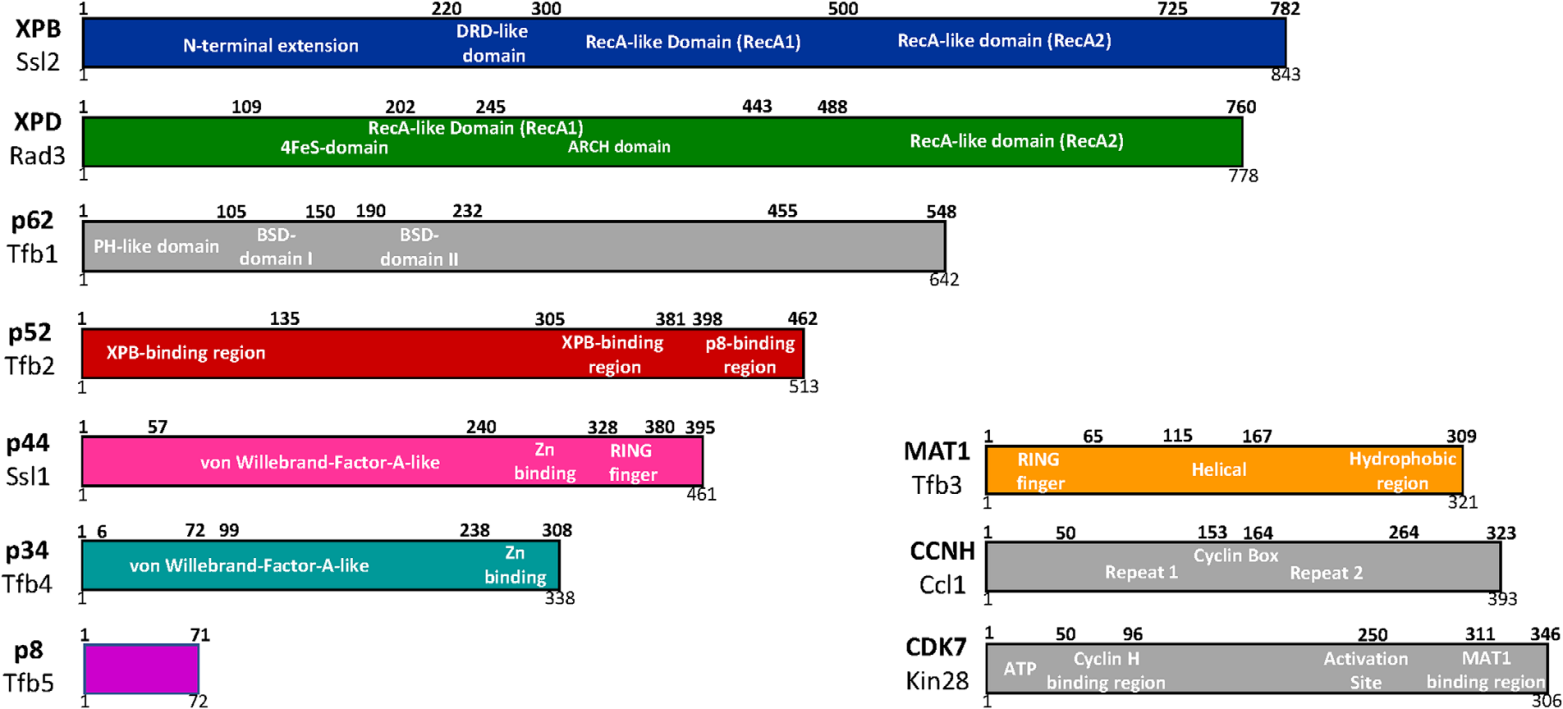
Domain organization of the TFIIH core (left) and CAK (right) subunits with some key domains highlighted. Human subunit names and residues are shown along the top for each subunit, with yeast subunits (*S. cerevisiae*) at the bottom.

The XPB and XPD subunits each contain two RecA‐like domains (ATPase/helicase); XPB has 3′‐5′ helicase activity whereas XPD has 5′‐3′ helicase activity. XPB also possesses a DRD‐like (DNA Damage Recognition Domain) domain in addition to an N‐terminal extension as depicted in Figure 1. XPB was initially characterized as a helicase,^12^ but can also function as a 5'‐3' DNA translocase,^13^, ^14^ at least with respect to its transcription‐associated roles (see below). The helicase activity of XPB is involved in the DNA damage response^15^ and works in conjunction with XPD.^5^, ^16^ In addition to its ATPase/helicase function enabled by the RecA‐like domains, XPD contains an ARCH domain and an iron‐sulfur cluster as shown in Figure 1. Basic roles in transcription (XPB) and DNA repair (XPD and XPB) are conserved going back to yeast.^16^, ^17^, ^18^, ^19^

Whereas XPB and XPD possess catalytic functions, additional subunits in the TFIIH core serve important structural and regulatory roles. The p62 subunit contains two BSD domains and a PH‐like (pleckstrin homology) domain (Fig. 1) that may mediate interactions with a variety of regulatory proteins, including sequence‐specific DNA‐binding transcription factors.^20^ The PH‐like domain also interacts with TFIIE,^21^, ^22^ which likely stabilizes TFIIH assembly within the PIC. The p52 subunit contains two XPB‐binding regions as well as a p8 binding region (Fig. 1) that helps stabilize the TFIIH core structure;^23^, ^24^ the Egly lab has shown that these interactions also stimulate XPB ATPase activity in both transcription and the DNA damage response.^24^, ^25^, ^26^ Through its interactions with p52 and XPB, the p8 subunit^27^, ^28^ stimulates XPB ATPase activity. Mutations in p8 or p52 that negatively affect the XPB–p52–p8 interaction network are associated with developmental disorders in humans and in model organisms.^23^, ^29^, ^30^

The p34 and p44 subunits are considered a structural “backbone” for TFIIH.^22^, ^31^ Both p34 and p44 contain a von Willebrand‐Factor‐A like domain and a Zn^2+^ binding region as depicted in Figure 1. Additionally, p44 contains a RING finger motif, which represents another Zn^2+^ binding region. The 5′‐3′ helicase activity of XPD is stimulated by p44, which directly interacts with XPD.^32^, ^33^ Notably, XPD mutations that are linked to developmental disorders XP and TTD inhibit the XPD‐p44 interaction and decrease XPD helicase and NER activity.^33^

### TFIIH CAK subunits: MAT1, CDK7, CCNH

Soon after its biochemical purification, TFIIH was identified as a factor that phosphorylates the pol II CTD.^6^, ^9^, ^10^, ^11^ The TFIIH kinase, CDK7 (Kin28 in *Saccharomyces cerevisiae*; Mcs6 in *Schizosaccharomyces pombe*), contains an N‐terminal CCNH binding region and a C‐terminal MAT1 binding region (Fig. 1).^34^ Collectively, the CDK7, CCNH, and MAT1 subunits form a stable kinase module that can reversibly associate with TFIIH.^11^, ^35^ In fact, CDK7–CCNH–MAT1 are known as the CDK Activating Kinase (CAK) complex in metazoans,^10^ based upon the ability of CDK7 to phosphorylate and activate cyclin‐dependent kinases. This role is not well conserved in yeast; the Cak1 protein performs this function in *S. cerevisiae* 36, 37, 38 whereas Csk1 and the CDK7 ortholog Mcs6 both serve as CAKs in *S. pombe*.^39^, ^40^

CDK7 activity and substrate specificity is regulated by CCNH and MAT1.^41^, ^42^, ^43^, ^44^, ^45^, ^46^ For example, CCNH controls substrate specificity toward CDKs involved in cell cycle regulation as well as the pol II CTD,^35^, ^47^ and MAT1 helps direct CDK7‐dependent phosphorylation toward DNA‐binding transcription factors.^48^, ^49^ MAT1 also stabilizes CCNH and CDK7 in the CAK complex^50^ and anchors it to the TFIIH core through interactions with XPD and XPB.^22^, ^51^, ^52^, ^53^

### TFIIH structure

**Figure 2:**
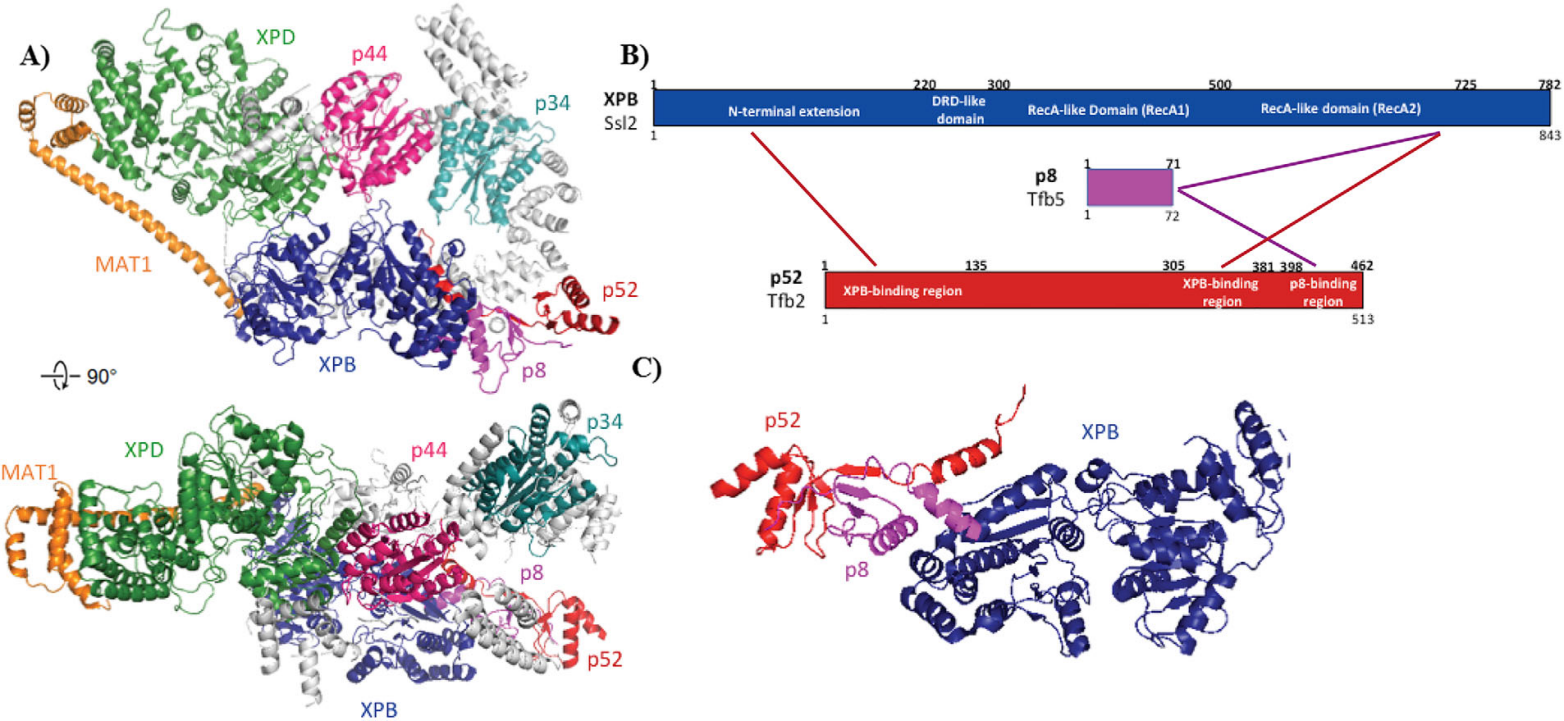
(A) CryoEM map of human TFIIH, with unassigned regions in grey (Ref. 34; PDB 5OF4). (B) Simplified scheme showing interactions/proximities between XPB with p8 and p52 from CXMS experiments with human and yeast TFIIH (Ref. 34). (C) Structural detail of p8–p52–XPB trimer. The XPB N‐terminal extension interacts with the N‐terminal region of p52; p8 binds the C‐terminal region of p52 and the C‐terminal region of XPB.

Building off pioneering work in other labs,^34^, ^54^, ^55^ the Nogales and Cramer groups have implemented the latest technological advances in cryoEM and single particle reconstruction techniques to advance our understanding of TFIIH structure and function.^22^, ^53^ The TFIIH core is horseshoe‐shaped; XPD (Rad3) and XPB (Ssl2) are at each end, connected by the other core subunits p44 (Ssl1), p34 (Tfb4), p52 (Tfb2), and p8 (Tfb5), as shown in Figure 2(A). Whereas the p62 (Tfb1) subunit could not be resolved in the human TFIIH structure,^53^ the Cramer group showed that for the yeast (*S. cerevisiae*) complex, Tfb1 extends along the horseshoe‐shaped surface, interacting with multiple core subunits.^22^

The recent cryoEM structural data for yeast and human TFIIH show a conserved subunit architecture,^22^, ^53^ in agreement with past studies.^34^ As expected, XPB (Ssl2) was shown to interact with p52 (Tfb2) and p8 (Tfb5) in both yeast and human TFIIH [Fig. 2(B)]. These findings were in agreement with biochemical and cellular experiments from the Egly lab that showed p52 interacts with and stimulates XPB ATPase activity.^24^, ^56^ Specifically, the XPB‐p52‐p8 heterotrimer results from interactions between the XPB RecA2 domain, the C‐terminal domain of p52, and p8 [Fig. 2(C)].^22^, ^34^, ^53^ The p8 mutation L21P likely destabilizes this interaction network, and this mutation is linked to TTD.^57^ The cryoEM data revealed structural details of previously characterized interactions between p52–XPB and p44–XPD and also established separate p34 structural interfaces with p52 and p44 that anchor the horseshoe‐shaped TFIIH core between XPD and XPB. The cryoEM^22^, ^53^ and crosslinking‐mass spectrometry data^34^ established that p34 and p44 dimerize through interaction between the Von Willebrand domain (vWA) of p34 and the RING finger domain of p44 (Fig. 1). This p34–p44 dimer may serve to seed assembly of the remaining core subunits, suggested by its interaction network with the other core subunits XPB, XPD, p62, and p52.^22^, ^31^, ^34^, ^53^

**Figure 3:**
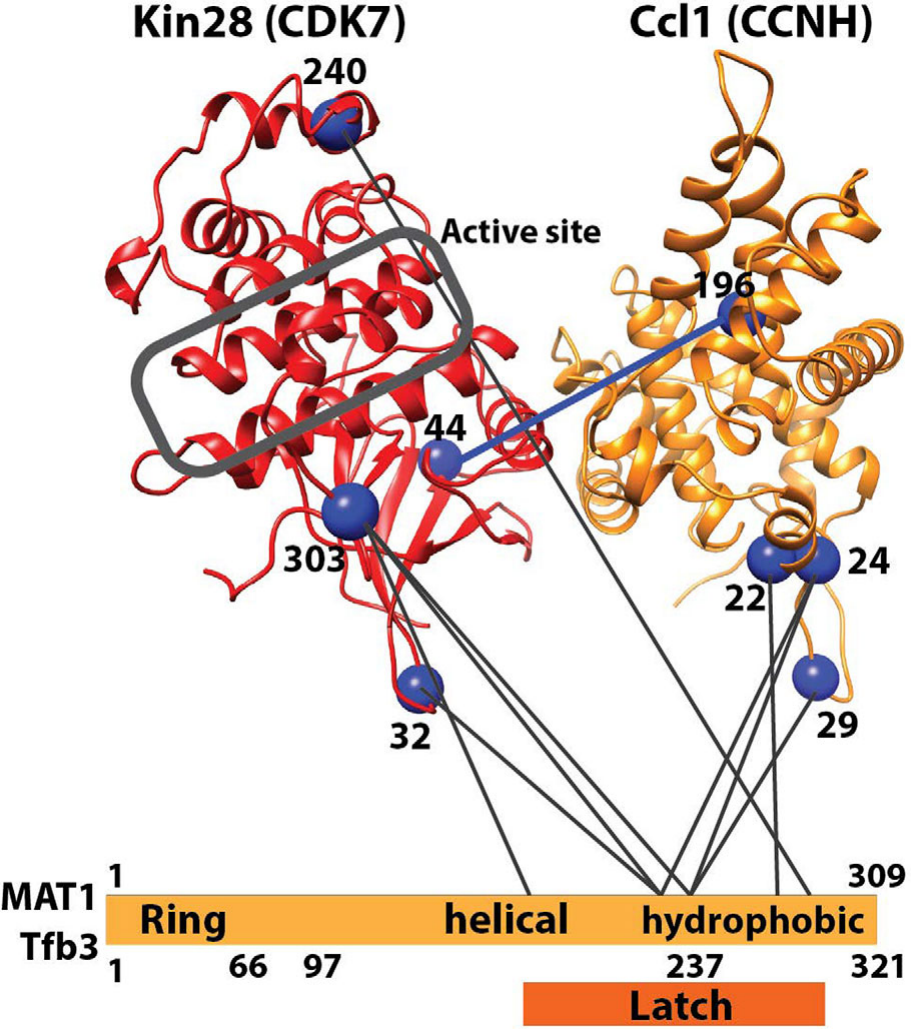
CXMS data mapped onto a yeast Kin28–Ccl1 (CDK7–CCNH) homology model and the Tfb3 (MAT1) sequence. Note the RING finger domain is not involved in these interactions as it binds between the pol II RPB7/POLR2G OB‐fold domain and the TFIIE E‐linker helices.

Whereas structural data reveal that XPB and XPD are located at each end of the horseshoe‐shaped core TFIIH complex [Fig. 2(A)], XPD and XPB also directly interact.^22^, ^34^, ^53^ Upon comparison of the free TFIIH structure with TFIIH within a partial PIC, the Nogales group noted evidence for a structural shift that breaks XPD–XPB contacts.^53^ MAT1, while not fully resolved in the cryo‐EM structures, was shown via CXMS to anchor the CAK to the core through interactions between its “latch” domain with XPB and XPD.^34^ This was corroborated by applying docking and homology models to the TFIIH cryoEM maps.^22^, ^53^ The XPD ARCH domain appears to interact with the MAT1 latch domain^34^, ^58^ and a C259Y mutation in the ARCH domain is linked to TTD; this mutation likely destabilizes the ARCH domain and may prevent proper CAK assembly with the TFIIH core. Although the CAK subunits MAT1 (Tfb3), CDK7 (Kin28), and CCNH (Ccl1) were largely unresolved in the cryoEM studies with human and yeast TFIIH, previous results have confirmed CDK7 interactions with MAT1 and CCNH, summarized in Figure 3.^34^, ^51^

## TFIIH and Transcription Regulation

**Figure 4:**
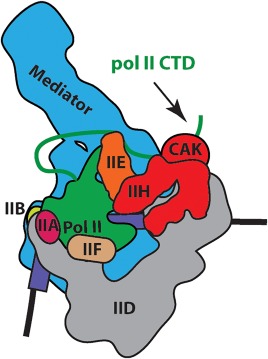
Schematic of the pre‐initiation complex (PIC), which consists of TFIIA, TFIIB, TFIID, TFIIE, TFIIF, TFIIH, Mediator, and RNA polymerase II (pol II).

**Figure 5:**
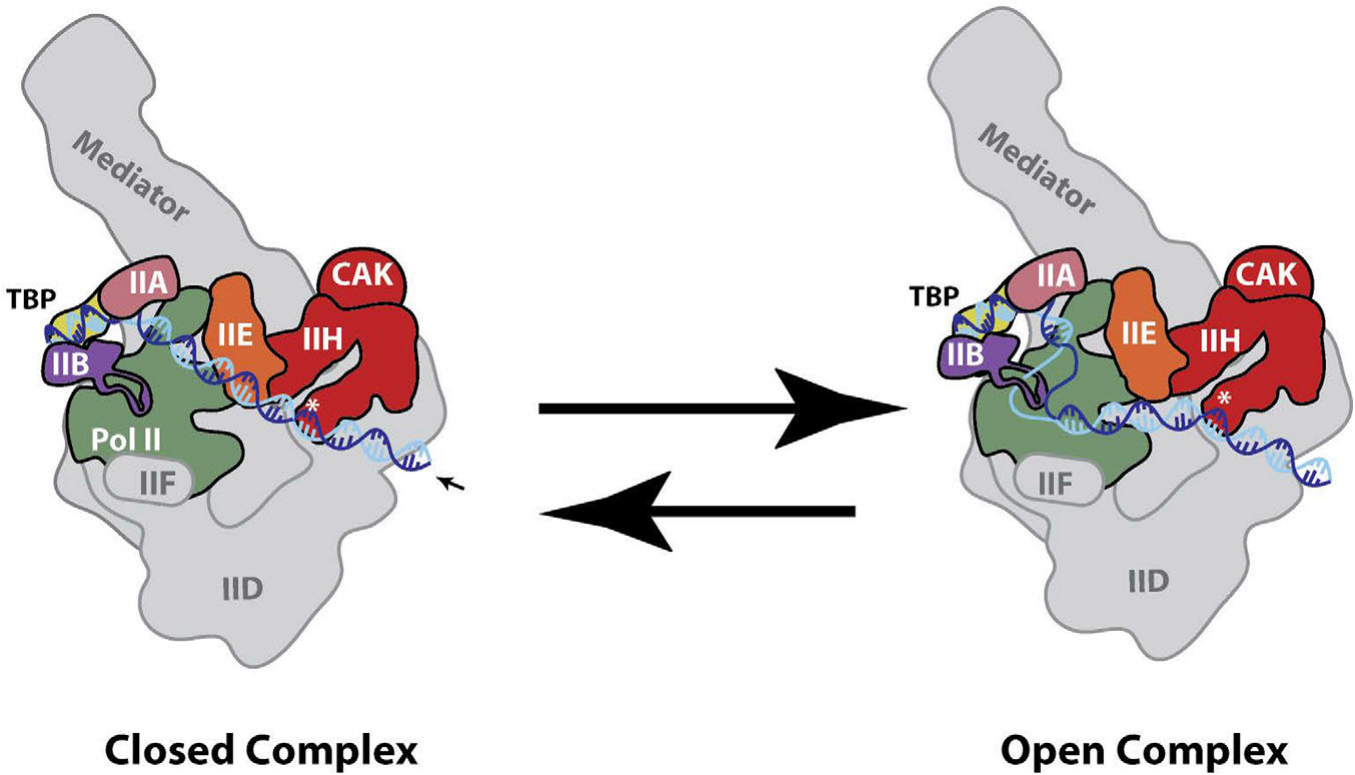
Schematic of XPB‐dependent promoter opening within PIC. XPB is denoted as an asterisk, which translocate in the 5'‐3' direction on the non‐template template strand (dark blue) to reel into the pol II cleft (arrow). Because upstream DNA is bound by TBP/TFIID, torsional strain is able to open promoter DNA (right). This open complex can re‐anneal to re‐generate the closed complex (left) or the open complex can be stabilized by PIC factors TFIIB, TFIIE, and TFIIF.

TFIIH is a basic component of the pol II transcription initiation machinery, commonly known as the pre‐initiation complex (PIC; see Fig. 4). In humans, the PIC is about 4.5 MDa in size and consists of TFIIA, TFIIB, TFIID, TFIIE, TFIIF, TFIIH, Mediator, and pol II. *In vitro*, the PIC can form in an ordered pathway that is likely relevant for *in vivo* assembly.^59^, ^60^, ^61^ TFIID, which contains the TATA‐binding protein TBP, first binds the TATA box upstream of the TSS; this pioneering event can nucleate assembly of TFIIA and TFIIB (which bind opposite ends of TBP), followed by TFIIF and pol II. Like TFIIF, TFIIE interacts directly with pol II,^61^, ^62^ and TFIIE binding helps assemble and orient TFIIH through multiple protein‐protein interfaces.^22^ As shown in Figure 5, TFIIH also directly contacts downstream promoter DNA, which helps anchor it in place within the PIC. Moreover, the Nogales and Cramer labs have shown that MAT1 (Tfb3 in *S. cerevisiae*) contacts the pol II stalk, providing rationale for the MAT1 requirement for transcription.^18^, ^22^ Although Mediator is not required to form a DNA‐bound complex containing TFIIA, TFIIB, TFIID, TFIIE, TFIIF, TFIIH, and pol II, Mediator stabilizes^61^ and regulates the entire PIC assembly, including TFIIH (see below).^63^

The Cramer lab determined a cryo‐EM structure of yeast (*S. cerevisiae*) TFIIH in the context of a minimal PIC, which provided important insights about how TFIIE and Mediator work together with TFIIH during transcription initiation. For instance, the structure revealed that Ssl2 (XPB) may be oriented on downstream DNA by the TFIIE E‐bridge helix; this interaction may also provide a physical basis for TFIIE‐stimulated DNA opening.^22^, ^64^, ^65^ It was also shown from the structural data that Rad3 (XPD) was located ∼40Å from the promoter DNA,^22^ in agreement with functional data that confirmed its helicase function was dispensable for pol II transcription.^66^ The kinase module of TFIIH (i.e. CDK7, CCNH, MAT1) is highly mobile and has been difficult to resolve in structural studies of the free TFIIH complex^53^, ^54^, ^59^ or in studies with minimal PIC assemblies.^22^, ^59^, ^62^ Notably, the TFIIH kinase module could not be resolved (i.e. too flexible/dynamic) in PICs lacking Mediator,^59^, ^62^ whereas the TFIIH kinase module could be resolved (albeit at limited resolution) within a yeast PIC that included a core Mediator complex.^22^ The TFIIH kinase module preferentially located between the “hook” and “shoulder” of Mediator within a yeast PIC.^22^ This location may be important for positioning Kin28 (CDK7) for pol II CTD phosphorylation, which is stimulated by Mediator.^67^, ^68^, ^69^, ^70^ However, the kinase module (a.k.a. the CAK) is likely not the only TFIIH interaction with Mediator;^71^ *T. brucei* contain a TFIIH‐like complex that lacks CAK homologs,^72^ yet a 7‐subunit core TFIIH complex forms a stable interaction with a *Trypanosoma brucei* Mediator complex.^73^

### Promoter opening

The TFIIH subunit XPB is arguably the most important for pol II transcription, as it contains an ATPase and translocase activity that enables ATP‐dependent opening of the promoter DNA at the transcription start site.^74^ This opening of the DNA template is required for transcription initiation; the single‐stranded “template” DNA can then descend into the cleft and engage the pol II active site. Moreover, promoter opening appears to represent an important regulatory stage for gene induction, at least in certain cell types or contexts.^75^ As shown schematically in Figure 5, XPB interacts with downstream DNA and uses its 5′‐3′ DNA translocase activity^13^, ^14^ to open promoter DNA, acting as a molecular wrench.^76^ Because upstream DNA is fixed through TBP/TFIID binding (which also bends the DNA), XPB 5'‐3' translocation along the non‐template strand (or 3'‐5' translocation on the template strand) would generate torsional stress that would be relieved by opening/melting the duplex DNA around the TSS. Thus, XPB acts to reel downstream DNA into the pol II cleft.^14^ The translocation mechanism for XPB has been most thoroughly studied with yeast TFIIH (XPB ortholog Ssl2), and biochemical data suggest Ssl2 enables DNA translocation in the 5'‐3' direction;^13^ in this case, translocation on the non‐template strand would open the promoter DNA. This XPB‐dependent “reeling” of DNA into the pol II cleft also helps explain why downstream DNA is required for TFIIH‐dependent stimulation of transcription *in vitro*.^77^

The XPB‐dependent reeling of DNA into the pol II cleft requires continuous ATP hydrolysis, and after short (∼10bp) translocation XPB dissociates from the DNA template;^13^ because TFIIH is anchored to the PIC through TFIIE and the pol II stalk,^22^, ^53^, ^62^ it is likely that XPB can rapidly re‐engage downstream promoter DNA as before. The melted template is unstable and can rapidly re‐anneal; however, PIC factors TFIIB, TFIIE, and TFIIF can interact with the template and non‐template strands to stabilize the open complex.^59^, ^61^, ^62^ Promoter re‐annealing at the TSS would re‐establish the PIC in the “closed” state, requiring renewed XPB function to generate the open complex. By contrast, a stable open template within the PIC can initiate transcription, but another barrier/regulatory checkpoint is promoter escape, which is described below.

In yeast, the TATA box can be variably spaced (i.e. greater than 30bp upstream of the TSS typical for human genes whose promoters contain TATA boxes). At yeast genes with TATA boxes greater than 30bp upstream of the TSS, TFIIH‐dependent promoter opening occurs upstream of the TSS; to ensure accurate initiation, pol II scans downstream sequences until the TSS is encountered. At that point, RNA synthesis is initiated. This process of TSS scanning occurs in a TFIIH‐ and XPB‐dependent manner but does not require RNA synthesis.^78^ However, TSS scanning in yeast appears to require continuous ATP‐hydrolysis and likely requires continuous translocation/reeling by the XPB/Ssl2 translocase.^13^, ^14^ Using single‐molecule approaches (optical tweezers), the Block and Kornberg groups showed evidence suggesting a large open bubble (average size: 85 bp with SNR20 promoter) as an intermediate in the TSS scanning mechanism.^79^ By contrast, data from the Galburt and Hahn labs supported a small (6 bp) open bubble during TSS scanning, followed by formation of a larger bubble (13 bp) upon transcription initiation.^80^ These contrasting models of TSS scanning may reflect differences in the experimental design; for instance, the large open complex (e.g. 85 bp single‐stranded DNA bubble) was observed upon applying larger forces (assistive or hindering) to the template DNA and the methods of PIC assembly were distinct in each case. Although XPB/Ssl2‐dependent TSS scanning does not appear to be important for human pol II transcription, it remains plausible that human TFIIH adopts similar scanning mechanisms as a nucleotide excision repair (NER) factor, to help localize the complex to DNA lesions.^81^

Interestingly, the Kornberg lab showed that, in a simplified transcription system, loss of the TFIIH kinase module (called TFIIK in *S. cerevisiae*) reduced TSS scanning and caused transcription to favor an “upstream” initiation site.^82^ These experiments lacked TFIID and could not be recapitulated in cells, suggesting auxiliary factors help enforce TSS scanning in *S. cerevisiae*. However, the data suggest new roles for the yeast kinase module (Tfb3, Ccl1, Kin28) in TFIIH‐dependent TSS scanning. This could be controlled by the Tfb3 subunit (homolog of human MAT1), which is highly flexible and tethers TFIIH to both TFIIE and the pol II enzyme;^22^ however, TSS scanning could also be enabled by Kin28 kinase activity. Kin28 substrates within the *S. cerevisiae* PIC include the pol II CTD and Mediator;^68^, ^83^ moreover, the Hahn lab has shown that Kin28 can promote ATP‐dependent (i.e. transcription‐independent) dissociation of the PIC to a re‐initiation‐competent scaffold complex.^83^ Whether such Tfb3‐ or Kin28‐dependent mechanisms underlie the link between TFIIK and pol II TSS scanning remain to be determined.

### Promoter escape and promoter‐proximal pausing

After formation of the open complex, pol II can initiate transcription but must break contacts with the PIC, in a process called promoter escape. Pol II promoter escape occurs after generation of a 12–13 base transcript and requires structural re‐organization of TFIIB.^84^, ^85^, ^86^ TFIIH contributes to promoter escape as well, through mechanisms involving XPB^87^ and CDK7‐dependent phosphorylation of the pol II CTD. The CTD of the RPB1/POLR2A subunit of human pol II contains 52 heptad repeats (26 in *S. cerevisiae*, 42 in *Drosophila*) of the general consensus sequence Y1‐S2‐P3‐T4‐S5‐P6‐S7.^88^ In its unphosphorylated form, the pol II CTD forms a high‐affinity^89^ interaction with Mediator.^68^, ^90^, ^91^ Pol II CTD phosphorylation by the TFIIH kinase CDK7 (which targets Ser5 and Ser7 in the CTD heptad repeats)^92^, ^93^, ^94^ releases Mediator interactions with the CTD,^95^, ^96^ which may help regulate promoter escape.^97^, ^98^

**Figure 6:**
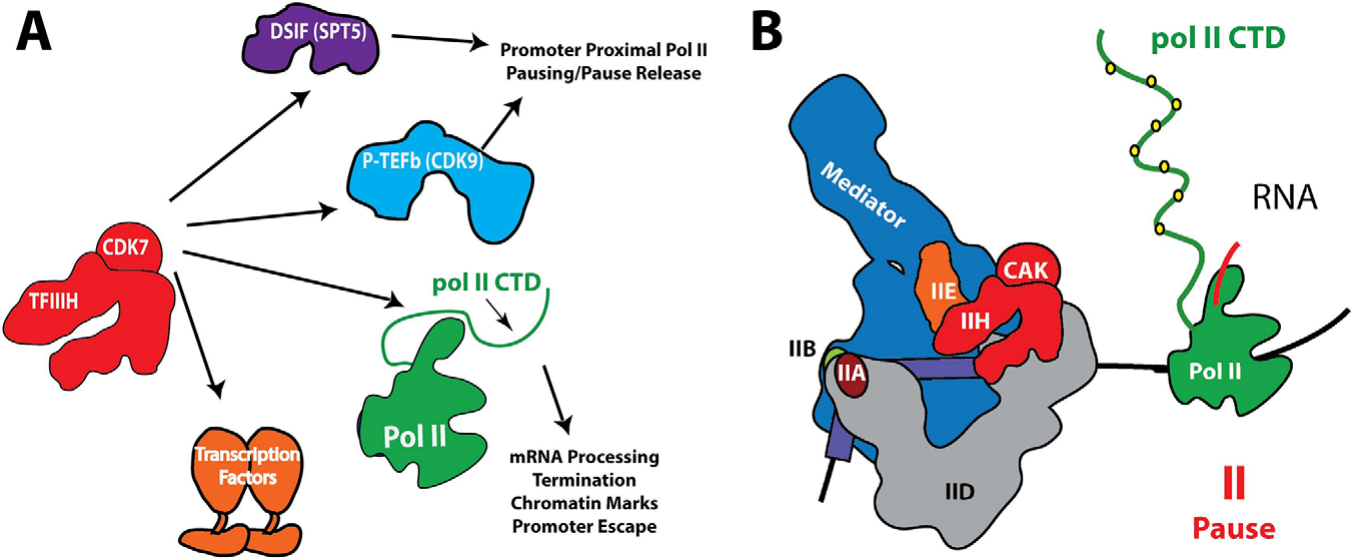
(A) Summary of TFIIH/CDK7 kinase targets that are implicated in regulation of pol II transcription. Whereas the pol II CTD and DSIF are common kinase targets for yeast and human TFIIH, CDK9 (P‐TEFb kinase) and DNA‐binding transcription factors are metazoan‐specific. (B) Following PIC assembly, promoter opening, initiation, and promoter escape, pol II typically pauses after transcribing less than 100 bases.

Following promoter escape, pol II typically pauses after generating a transcript of 20–80 bases in length (Fig. 6).^99^ Although promoter‐proximal pol II pausing is widespread in metazoans, it is not uniformly observed in yeast.^100^ Paused pol II complexes are largely lacking in the yeast *S. cerevisiae*, whereas paused intermediates are observed in *S. pombe*.^101^ In human cells, it has been shown that selective inhibition of the TFIIH‐associated kinase, CDK7, increases pol II promoter‐proximal pausing at thousands of genes under normal growth conditions.^96^ The DSIF (DRB sensitivity inducing factor) complex, which consists of SPT4 and SPT5, is present throughout eukaryotic lineages and is a well‐established regulator of promoter‐proximal pol II pausing.^102^ DSIF associates with pol II after promoter escape; in fact, DSIF interactions with pol II are occluded by PIC factors TFIIB, TFIIE, and TFIIF.^103^ The Gilmour lab has shown that DSIF–pol II interaction can be stabilized by nascent RNA emerging from the pol II exit channel^104^ and structural data from the Cramer lab has implicated an upstream (i.e. behind transcribing pol II) “DNA clamp” and an “RNA clamp” adjacent to the RNA exit channel as potential domains important for regulation of pol II pausing.^103^

The parallels between DSIF and CDK7 in the regulation of pol II activity are striking. CDK7 (Kin28 in *S. cerevisiae*) phosphorylates SPT5^42^, ^45^ and this may control DSIF‐dependent regulation of pol II pausing (Fig. 6). Inhibition of CDK7 also reduces DSIF recruitment to promoter‐proximal regions.^105^, ^106^ Depletion of SPT5 in human cells caused defects in RNA processing (e.g. defective capping and splicing) similar to those observed upon CDK7 inhibition.^107^ Moreover, Spt5 depletion in murine embryonic fibroblasts (MEFs) caused defects in pol II processivity (e.g. premature termination on long genes) that roughly approximated processivity defects observed in yeast upon inhibition of Kin28.^108^, ^109^ In *S. cerevisiae*, Spt5 contributes to capping enzyme recruitment^110^ and inhibition of Kin28 caused pol II accumulation near the +2 nucleosome,^109^ similar to degron‐depleted Spt5 cells.^111^ Moreover, in *S. pombe*, Spt5 protein levels were shown to be sensitive to the kinase activity of its CDK7 ortholog Mcs6 (increased Spt5 protein upon Mcs6 inhibition).^112^ Collectively, these results suggest a conserved functional co‐dependence between the TFIIH kinase and DSIF.

Another well‐established regulator of pol II promoter‐proximal pausing is the four‐subunit NELF complex, which is conserved in metazoans but is absent in yeast genomes. NELF may act in concert with DSIF to stabilize pol II pausing,^99^, ^102^ and DSIF and NELF recruitment appears to be regulated by CDK7 kinase activity;^93^, ^105^, ^106^ however, the mechanistic basis for these processes remains unclear. Human CDK7 also phosphorylates and activates CDK9 [Fig. 6(A)],^105^ the P‐TEFb kinase that is broadly implicated in the regulation of pol II pausing and pause release.^113^ Like CDK7, activated CDK9 can also phosphorylate DSIF,^114^ which may facilitate pol II pause release into productive elongation.

Additional mechanisms by which CDK7 appears to impact promoter‐proximal pausing are through its phosphorylation of the pol II CTD. Whereas unphosphorylated CTD interacts with the Mediator complex, TFIIH phosphorylation triggers a new set of CTD interactors, including RNA capping enzymes.^96^, ^112^, ^115^, ^116^, ^117^, ^118^ Pol II pausing may have evolved in part to ensure that nascent transcripts are appropriately 5'‐capped prior to the transition to productive elongation.^119^, ^120^ TFIIH‐dependent phosphorylation of the pol II CTD also enhances PAF1 complex association,^96^ and the PAF1 complex has been shown by the Roeder and Shilatifard labs to control pol II promoter‐proximal pausing in human cells.^121^, ^122^, ^123^

### Elongation, RNA processing, and termination

The TFIIH‐associated kinase CDK7 (Kin28 in *S. cerevisiae*) influences pol II transcription beyond the promoter‐proximal region, via phosphorylation of the pol II CTD. In yeast and mammalian cells, TFIIH‐phosphorylated CTD both recruits^96^, ^106^, ^116^, ^117^, ^118^, ^124^, ^125^, ^126^, ^127^ and activates^115^ 5'‐capping enzymes for the nascent RNA transcript. Moreover, Pol II CTD phosphorylation has been shown to affect pol II elongation rates, and elongation rates, in turn, have been shown to affect alternate splicing in mammalian cells.^128^, ^129^, ^130^ As a major pol II CTD kinase, CDK7 likely impacts pol II elongation in this way. In agreement, pol II CTD phosphorylation, including at Ser5 (a primary CDK7 target), has been shown to be important for splicing regulation,^131^ and pol II pausing in gene bodies correlates with splicing, providing further evidence for a transcription rate effect.^132^

CDK7 activity is also linked to pol II termination at gene 3'‐ends. Inhibition of CDK7 causes termination defects in yeast and human cells.^93^, ^96^, ^105^, ^133^ However, the termination defects appear to manifest in mechanistically distinct ways in human and yeast cells. Increased read‐through transcription was observed in human cells upon CDK7 inhibition^93^, ^96^, ^105^ whereas premature termination was seen upon inhibition of the CDK7 ortholog Kin28 in *S. cerevisiae*.^109^, ^134^ Because human CDK7 activates the P‐TEFb kinase CDK9 (another major pol II CTD kinase),^105^ inhibition of CDK7 has amplified effects on CTD phosphorylation, and this may contribute to the distinct effects of CDK7 inhibition in yeast versus human cells.

The defects in pol II elongation (*S. cerevisiae*) and termination (human) upon inhibition of TFIIH kinase activity may also reflect TFIIH‐dependent phosphorylation of SPT5, which is a conserved regulator of pol II elongation.^135^ However, because few high‐confidence targets of CDK7 have been identified in yeast or mammalian cells, it remains likely that other substrates contribute to the elongation, RNA processing, and termination defects observed upon inhibition of TFIIH kinase activity.

### CDK7 as an epigenetic regulator of pol II transcription

Studies in yeast and mammalian cells have revealed that phosphorylated forms of the pol II CTD preferentially interact with H3K4 and H3K36 methyltransferases.^136^, ^137^, ^138^, ^139^ Comparative proteomics of factors bound to TFIIH‐ versus P‐TEFb‐phosphorylated pol II CTD implicated the H3K36 methyltransferase SETD2 as binding specifically to P‐TEFb‐phosphorylated CTD, whereas the H3K4 methyltransferase complexes SETD1A/B interacted with phosphorylated CTD more generally (i.e. TFIIH‐ or P‐TEFb‐phosphorylated).^96^ Because CDK7 activates the P‐TEFb kinase CDK9 in human cells,^105^ these results implicate CDK7 as an epigenetic regulator of transcription‐associated chromatin marks. In agreement, targeted inhibition of CDK7 in human cells decreased H3K4me3 spreading into gene bodies and altered the distribution of H3K36me3 toward gene 3'‐ends.^96^

Although the term “epigenetic” has taken on various meanings,^140^ a strict definition requires a heritable trait that does not result from a change in genomic DNA sequence. DNA CpG methylation is considered heritable,^141^ and emerging evidence suggests histone marks can also be heritable.^142^, ^143^, ^144^ The transcription‐associated histone marks that are affected by CDK7 kinase activity, H3K4me3 and H3K36me3, are each linked to DNA methylation. H3K4me3 marks are concentrated in regions lacking DNA methylation,^145^ whereas H3K36me3 levels directly correlate with DNA (CpG) methylation.^146^ In this way, the TFIIH‐associated kinase CDK7 may indirectly control DNA methylation patterns in mammalian cells, with potential consequences for regulation of gene expression patterns in cell progeny. Incidentally, the histone marks affected by human CDK7 activity (H3K4me3 and H3K36me3) have also been shown to influence splicing.^147^, ^148^, ^149^

### CDK7 as a potential regulator of DNA‐binding TFs

Sequence‐specific DNA‐binding TFs regulate all physiological processes; CDK7 is known to phosphorylate numerous DNA‐binding TFs, including p53^150^ and nuclear receptors.^151^, ^152^, ^153^, ^154^ Not all phosphorylation sites are functionally relevant, however,^155^ and it remains to be determined how TFIIH‐dependent TF phosphorylation directly controls TF function.

## Regulators of TFIIH Function: TFIIE and Mediator

TFIIE is a well‐established regulator of TFIIH; TFIIE helps recruit TFIIH to the promoter and both TFIIE and TFIIH enable promoter opening.^65^, ^156^, ^157^ A physical basis for recruitment was observed from recent cryoEM data from the Cramer lab, which showed four separate contacts between TFIIE and TFIIH within a minimal yeast PIC.^22^ An “E‐bridge” helix contacts XPB (Ssl2), and p62 (Tfb1) contacts TFIIE through separate “E‐floater” and “E‐dock” helices. Specifically, the E‐dock interacts with the p62 (Tfb1) PH‐like domain, the E‐floater contacts p62 (Tfb1) through its BSD1 domain, and the E‐bridge helix connects the p62 (Tfb1) BSD2 domain to XPB (Ssl2) lobe 2.^22^ The E‐bridge interaction with p62 (Tfb1) and XPB (Ssl2) may influence conformational ratcheting, supporting a role for TFIIE‐dependent stimulation of DNA opening. A TFIIE–TFIIH interaction was also observed through XPD (Rad3) in the yeast minimal PIC structure,^22^ at the base of the pol II stalk, in agreement with cryoEM data with human factors.^59^, ^62^

Mediator has also been shown to regulate TFIIH function. Mediator directly interacts with TFIIH^22^, ^71^ and stimulates CDK7 kinase activity.^68^, ^69^, ^70^ Structural data from the Kornberg and Cramer labs reveal direct binding between the yeast TFIIH CAK and the Mediator hook, knob, and shoulder domains,^22^, ^89^ implicating these interactions in CDK7 (Kin28) activation. Because Mediator binds with high affinity to the unphosphorylated pol II CTD,^89^, ^90^ Mediator may also position the CTD favorably for CDK7‐dependent phosphorylation.^22^, ^89^, ^158^ Pol II CTD phosphorylation releases its interaction with Mediator,^95^, ^96^ which may contribute to pol II promoter escape.^97^, ^98^ The Mediator‐associated kinase CDK8 (Srb10 in *S. cerevisiae*) also appears to regulate CDK7 (Kin28) function through mechanisms that remain unclear.^83^, ^159^ Moreover, biochemical experiments from the Malik group implicate Mediator‐dependent regulation of TFIIH, perhaps through activation of XPB.^160^

## Cellular Roles for TFIIH Beyond Transcription

Subunits of TFIIH have been linked to important cellular functions that do not involve pol II transcription. Several of these functions are described in the following sections.

### DNA repair

The TFIIH core subunits XPD and XPB both mediate the response to DNA damage and structural interactions between the CAK and TFIIH core subunits serve to regulate XPD and XPB function during nucleotide excision repair (NER). The helicase activity of XPD is essential for its NER function,^161^ and C‐terminal mutations of XPD that destabilize its interactions with p44 reduce its NER activity.^33^ The TFIIH CAK subunit MAT1 negatively regulates the XPD ATPase/helicase,^58^ and structural data reveal a direct interaction between MAT1 (Tfb3 in *S. cerevisiae*) and XPD (Rad3 in *S. cerevisiae*) that likely enables this regulation.^22^, ^34^, ^53^ Removal of the CAK thus activates XPD for unwinding the DNA around the lesion site. A DNA repair factor that performs this task is XPA.^18^, ^25^ Incidentally, MAT1 and CCNH each contribute to the stability of the CAK, ensuring it remains stable (and CDK7 active as a kinase) apart from the TFIIH core. A role for XPB in nucleotide excision repair remains enigmatic, although its ATPase/helicase function appears to be important for TFIIH localization to the site of DNA damage.^81^ These complementary roles for XPB and XPD facilitate recognition and enable repair via other factors (reviewed in refs. 1–3).

### Additional roles for XPB and XPD: retroviruses and G‐quadruplexes

The TFIIH core subunits XPB and XPD have been found to protect against retroviral insertion into the host genome. This occurs through degradation of retroviral cDNA;^162^ XPB or XPD mutants deficient in NER activity had higher rates of viral integration and these effects did not appear to result from changes in transcriptional activity.^162^, ^163^ Thus, it appears that XPB and XPD act not only to detect and initiate repair of DNA damage, they also serve to safeguard the host against retroviral infection. The correlation between NER‐deficient XPB or XPD and viral genome integration suggests that retroviral insertion favors sites of unrepaired DNA damage.

ChIP‐Seq data in human cells have shown that XPD and XPB genomic occupancy overlaps with sequences predicted to form G‐quadruplex structures,^164^ which are four‐stranded helical structures that can form in GC‐rich genomic regions.^165^ Additional biochemical experiments suggested that whereas both XPB and XPD can bind G‐quadruplex DNA, only XPD is capable of unwinding these structures. These biochemical assays were completed with single‐subunit XPB and XPD homologs from thermophilic microbes, however, and it remains to be seen whether similar activities are recapitulated within the context of the entire TFIIH complex.^164^ These results suggest additional functions for the TFIIH XPB and XPD subunits that may expand upon and/or contribute to their established roles in pol II transcription and DNA repair.

### Cell cycle regulation

In mammals, the TFIIH CAK subunits (MAT1, CDK7, CCNH) are essential for cell cycle regulation. For example, loss of MAT1 in mice caused defects in S phase entry^166^ and inhibition of CDK7 functions similarly by preventing CDK1 and CDK2 activation in human cells.^167^ The CAK regulates the cell cycle through T‐loop phosphorylation and activation of CDK1, CDK2, CDK4, and CDK6.^168^, ^169^, ^170^ Whereas its role in cell cycle regulation is not conserved in *S. cerevisiae*, the CDK7 ortholog in *S. pombe*, Mcs6, does control cell cycle progression, in coordination with the Lsk1 kinase.^112^, ^171^ Taken together, these findings reveal that whereas the complete TFIIH complex (i.e. core + CAK) is required for transcriptional activity, the CAK is important for cell cycle regulation.

### Neurogenesis and memory formation

Recent studies in model organisms have linked the TFIIH kinase CDK7 to neurogenesis and long‐term memory formation. In the developing mouse neocortex, Cdk7 expression was observed to correlate with miR‐210 expression, which targets Cdk7. Consistent with a role for miR‐210 in regulation of Cdk7 levels throughout neuronal development, decreased Cdk7 (or increased miR‐210) promoted differentiation of neural progenitors to post‐mitotic neurons.^172^ Reduced or elevated Cdk7 levels also had predictable effects on cell cycle progression and proliferation of neural progenitor cells (see above).^172^ These findings are in general agreement with links between Cdk7 in mouse development and stem cell maintenance.^173^ A study in *C. elegans* has also connected Cdk7 activity to neuronal differentiation, suggesting ancient links to neurogenesis.^174^ In a mouse model study of post‐mitotic neurons, He et al. observed that Cdk7 expression was increased compared with developing neurons, and that Cdk7 inhibition (with THZ1) impaired long‐term memory formation, whereas short‐term memory was unaffected.^175^ Collectively, these findings correlate CDK7 activity to neuronal development and function; however, these links likely reflect, at least in part, the key requirement for CDK7 in pol II‐dependent gene expression. Memory formation requires new transcription (e.g. of immediate early genes, many of which are DNA‐binding TFs), and these findings with CDK7 are reminiscent of other studies that have linked general regulators of pol II transcription to memory formation in mammals.^176^

## Pathologies Associated with TFIIH Function

Defects in TFIIH function are linked to developmental diseases and numerous cancers (Table 2), and TFIIH is also targeted by several viral pathogens. These are summarized below.

**Table 2:** Pathologies Associated with TFIIH Core Subunits XPB, XPD, and p8 as Well as Those Associated with the CAK Subunit CDK7

TFIIH subunit	Associated pathologies
XPB	Xeroderma pigmentosum (XP), XP combined with Cockayne Syndrome (XP/CS), Trichothiodystrophy (TTD)
XPD	Xeroderma pigmentosum (XP), XP combined with Cockayne Syndrome (XP/CS), Trichothiodystrophy (TTD)
p8	Trichothiodystrophy (TTD)
CDK7	Triple Negative Breast Cancer, Peripheral T‐Cell Lymphomas, Ovarian Cancer

### Developmental diseases

The developmental diseases Xeroderma Pigmentosum (XP), Cockayne Syndrome (CS), and Trichothiodystrophy (TTD) are associated with mutations in XPB, XPD, and p8. These mutations negatively affect a range of TFIIH functions in transcription and/or NER.^57^ However, TTD‐associated mutations typically affect TFIIH transcriptional activity whereas XP and XP/CS are typically associated with NER deficiencies. Although XP, CS, and TTD have distinct symptoms, each results from recessive mutations and share UV sensitivity.^177^ As shown in Table 3, XPD mutations are more numerous compared with XPB or p8 mutations identified in the clinic.

**Table 3:** Missense Mutations in the TFIIH Core Subunits XPB, PD, and p8 Associated with the Neurological and Developmental Disorders XP, XP/CS, and TTD

TFIIH subunit	XP	XP/CS	TTD
XPB	R425STOP	F99S, Q545STOP	T119P
XPD	R683W (+ others)	D681H	R112H, C259Y, R658C/H, R722W (+ others)
p8	—	—	M1P, L21P, R56STOP

XP is an autosomal recessive disorder resulting in skin abnormalities and neurodegeneration. The UV sensitivity associated with XP greatly increases the chance of melanoma and squamous and basal cell carcinomas.^177^ XP is associated with mutations in XPD and XPB, as well as other proteins that are not TFIIH subunits. As shown in Table 3, the missense mutation in XPB (R425STOP) results in XP phenotypes along with other frameshift mutations. The R683W XPD mutation represents a common XPD site associated with XP, although more than 30 other XPD mutations have been identified in the clinic. XP patients show deficient XPD helicase activity, consistent with defects in NER.^178^

CS is a recessive disease that varies in the severity of symptoms, but typically is associated with developmental and neurological disorders. The mildest form of CS, called UVSS, manifests as mild photosensitivity. More moderate forms, called CSI and CSII, exhibit a multitude of symptoms including reduced lifespan (roughly 12 years), dwarfism, retinopathy, microencephaly, and other developmental defects. The most severe form of CS, called COFS, is neonatal lethal.^177^ Mutations in XPB and XPD also contribute to XP/CS, a condition with overlapping symptoms of XP and CS.^57^ Symptoms include skin abnormalities associated with XP and severe neurological and developmental defects associated with CS.^57^ As shown in Table 3, the XPB missense mutations F99S and Q545STOP and the XPD missense mutation D681H are commonly associated with XP/CS; however, other frameshift mutations in these subunits have been documented. These XPB and XPD mutations may prevent CAK module dissociation that is required for NER or could block normal function of other DNA repair factors to cause delayed transcriptional resumption (i.e. after DNA damage) commonly seen in XP/CS patients.^179^, ^180^, ^181^

TTD is an autosomal recessive disorder with symptoms including brittle hair and ichthyosis. Those with TTD may also have reduced lifespan and mild to severe mental retardation. TTD is associated with mutations in XPB, XPD, or p8 (Table 3), with many mutants identified in XPD, including missense and frameshift mutations.^177^, ^182^, ^183^ TTD is associated with an overall decrease in cellular TFIIH concentration and basal transcriptional defects.^178^, ^183^

### Viral pathogenesis

TFIIH is targeted by several proteins expressed by pathogenic viruses.^184^ For example, the NSs protein expressed by the Rift Valley Fever Virus (RVFV) was shown by the Egly lab to bind the TFIIH subunit p44, resulting in overall loss of TFIIH function.^185^ Other labs later showed that the RVFV NSs protein can similarly target and degrade TFIIH subunits such as p62.^186^, ^187^ Moreover, the HIV Tat protein binds the TFIIH CAK and activates CDK7 to promote expression of the viral genome.^188^, ^189^ TFIIH recruitment to the HIV‐1 promoter was also shown to be an essential, late‐stage event required for HIV emergence from a latent state.^190^ These TFIIH links to viral pathogenesis are underscored by the finding that CDK7 inhibition has broad‐spectrum antiviral activity, blocking replication of cytomegaloviruses, herpesviruses, and adenoviruses in human cells.^191^

### Cancer

Numerous cancers appear to be dependent on elevated CDK7 activity to drive their oncogenic state.^192^, ^193^ Because of its role in cell cycle regulation, CDK7 activity cannot be entirely de‐coupled from its broad transcriptional effects. However, elevated expression of oncogenes is enabled by clusters of enhancers, or super‐enhancers,^194^, ^195^, ^196^ that require CDK7 activity to maintain their high‐level expression.^197^ Consequently, these genes are especially sensitive to CDK7 inhibition. Small cell lung cancers, triple negative breast cancer, and T‐cell acute lymphoblastic leukemias are each aggressive cancers with high mortality rates; notably, each is sensitive to THZ1, a covalent inhibitor of CDK7.^198^, ^199^, ^200^, ^201^ As expected, CDK7 inhibition by THZ1 is broadly cytotoxic,^200^ and although THZ1 inhibits other kinases, it represents a powerful means to assess potential roles for CDK7 kinase activity in cancer cell proliferation. Indeed, THZ1 has revealed numerous transcriptional dependencies in cancer cell lines that suggest novel combinatorial approaches (e.g. CDK7 inhibition + conventional therapeutic) for cancer treatment.^202^, ^203^ Furthermore, pre‐clinical studies have shown that THZ1 can inhibit the emergence of multi‐drug resistance in human cells and mouse models.^204^ A biological rationale for these findings is that drug resistance requires adaptive transcriptional reprogramming that is blocked by persistent, low‐level inhibition of CDK7.^204^ These and other results provide a promising proof‐of‐concept that further development of CDK7 inhibitors could yield therapeutic strategies that will be effective in the clinic.

## Small Molecule Inhibitors of TFIIH Function

Although other small molecule inhibitors of CDK7 have been described,^191^, ^203^, ^205^ the most widely used has been THZ1, developed in the lab of Nathaneal Gray.^200^ THZ1 is an ATP analog that contains an extension with a Michael acceptor that is positioned to react with C312, which is adjacent to the CDK7 ATP binding site. As noted above, THZ1 has served as a valuable tool to probe the role of CDK7 in mammalian cells; however, although it is most potent against CDK7, it also inhibits dozens of other kinases. Consequently, experiments with THZ1 must be interpreted with caution.

Chemical genetics methods, pioneered by the Shokat lab,^206^ have been successful in selective inhibition of CDK7 activity in yeast and human cells.^42^, ^83^ This strategy requires a “gatekeeper” mutation (e.g. F91G in human CDK7) in the ATP binding pocket that will accommodate a bulky ATP analog such as NM‐PP1. In this way, normal kinase function is retained; however, in the presence of NM‐PP1 (which cannot bind native kinases and therefore does not compete with ATP), the mutant CDK7 allele is selectively inhibited. The Ansari lab has advanced this concept to design a covalent inhibitor of yeast CDK7, Kin28.^109^ Studies that have implemented these “chemical genetic” strategies have markedly advanced our understanding of the roles of the TFIIH kinase in transcription; however, because genome editing is required, these approaches cannot be easily translated to the clinic.

Triptolide is a highly selective inhibitor of XPB that covalently binds at Cys 342 to inhibit its ATPase activity.^207^, ^208^ Importantly, an XPB C342T mutant was shown to be resistant to triptolide treatment, confirming XPB as the relevant cellular target.^207^ Triptolide is a potent inhibitor of pol II transcription (IC_50_ of 12nM at 12 hours of treatment^208^); however, numerous labs have noted pol II degradation is induced with prolonged triptolide treatment times.^209^, ^210^, ^211^ Thus, triptolide treatment must be brief to reliably assess its effects on pol II transcription. Using a short (1 hour) triptolide treatment in human cells (HCT116), the Shilatifard lab showed that transcription of most pol II genes was inhibited, but a small subset were resistant.^209^ This suggested that this subset of genes was either stably paused or may not be dependent upon XPB for activation. Similar results were seen by the Lis lab in triptolide‐treated murine ES cells.^212^ Using a nascent RNA technique called GRO‐Seq,^213^ the Lis lab noted that, as expected for an XPB inhibitor, triptolide caused genome‐wide loss of new transcription and promoter reads over time.

Another small molecule that targets XPB is spironolactone, which appears to selectively degrade the XPB protein while retaining the structural integrity of the rest of the TFIIH complex.^214^ This led to the provocative conclusion that, contrary to existing models, XPB function was not generally required for pol II transcription in human cells.^215^ Because XPB requires continual ATP hydrolysis to maintain an open DNA template,^13^, ^14^, ^64^ these findings suggested a unified mechanism of promoter opening from bacteria to mammalian cells that does not require ATP hydrolysis. Whereas other studies have suggested that TFIIH/XPB function may not be required at all pol II‐transcribed genes,^61^, ^209^, ^216^ the data with spironolactone were the first to suggest XPB function was not generally required for pol II transcription in human cells.^215^ The spironolactone data should be interpreted with caution, however, and not simply because they contradict well‐tested models of pol II open complex formation in eukaryotic cells. Spironolactone is a reactive compound and possesses functional groups (e.g. Michael acceptor) that are designated as PAINS (Pan‐Assay INterference compoundS) by the medicinal chemistry community.^217^ This is corroborated by its wide range of physiological effects in the clinic. Based upon structural data and mutagenesis experiments, XPB itself appears to be important for maintaining the integrity of the TFIIH complex,^22^, ^34^, ^53^, ^218^ and thus it seems unlikely that XPB could be selectively extracted and degraded without negatively affecting TFIIH structural integrity. It is notable, however, that evidence in yeast suggests that XPB (Ssl2) may be labile, dependent upon the Tfb6 protein,^219^ which lacks a clear ortholog in human cells.

## Conclusions and Outstanding Questions

Many basic cellular functions for TFIIH have been defined, and its known links to human disease are extensive and are certain to expand. Below we highlight a few, among many, interesting and outstanding questions.

- How does TFIIH function within the entire PIC during pol II initiation, promoter escape, and promoter‐proximal pausing? Because the TFIIH subunit XPB binds DNA downstream of the TSS, pol II must bypass XPB to transition to productive elongation. TFIID also binds DNA downstream of the TSS,^220^ and functional interactions between TFIIH and TAF7 have been described.^221^ Does TFIID help regulate this process? The multiple interactions between TFIIH and TFIIE^22^ likely tether TFIIH to a scaffold PIC that remains after pol II promoter escape;^222^ however, a structural and mechanistic understanding of this re‐initiation intermediate is lacking.
- Is XPB required for open complex formation at all genes? Under what circumstances might XPB/TFIIH not be required? *In vitro* assays have demonstrated that TFIIH is not required for open complex formation if the DNA template is negatively supercoiled.^65^ Antisense transcription (i.e. on the non‐template strand, transcribing in the opposite direction) is widespread in mammalian cells^213^, ^223^ and would promote negative supercoiling at the promoter. Potentially, this could preclude XPB action during pol II transcription initiation at some genes.
- How does TFIIH function during DNA repair? The PH‐like domain of p62 has been shown to interact with several DNA repair factors^224^, ^225^ in a process that is regulated in part by the chromatin remodeler CHD1.^226^ How are these interactions controlled? How does TFIIH signal other repair factors to mobilize to sites of DNA damage?
- The ATPase/helicase/translocase function for the TFIIH subunits XPB and XPD is implicated in a variety of cellular processes. What range of substrates do XPB or XPD act upon? Could XPB or XPD unwind RNA structures or help resolve R‐loops? Novel TFIIH‐associated factors continue to be identified.^227^ Do these auxiliary factors alter XPB or XPD function?
- What are the most functionally relevant substrates for CDK7? Whereas several key substrates have been identified that support its role in cell cycle regulation, few transcription‐related targets are known beyond CDK9, SPT5, and the pol II CTD. A first step in understanding the cellular roles of any kinase is to identify its substrates. If this can be accomplished, the next challenge will be to determine the functional consequences of specific phosphorylation events.
- Will improved inhibitors of TFIIH be developed? Whereas small molecule inhibitors are likely to remain widely used as molecular probes for basic science research, how will TFIIH inhibitors fare in the clinic?

Progress toward answering these and other questions will continue to rely on improved biophysical, chemical, and cellular tools (e.g. transcriptomics). Mechanistic insights will require structural and *in vitro* ensemble and single‐molecule approaches that should be able to parse out key structural and functional intermediates. As in previous decades of TFIIH research, clinical identification and biochemical characterization of disease‐associated mutants will also advance understanding and may yield new strategies for molecular therapeutics.

## Conflict of Interest

The authors have no conflict of interest to declare.

## References

[1] Compe E , Egly JM (2016) Nucleotide excision repair and transcriptional regulation: TFIIH and beyond. Annu Rev Biochem 85:265–290. 27294439 10.1146/annurev-biochem-060815-014857

[2] Houten BV , Kuper J , Kisker C (2016) Role of XPD in cellular functions: to TFIIH and beyond. DNA Repair 44:136–142. 27262611 10.1016/j.dnarep.2016.05.019

[3] Spies M (2014) Two steps forward, one step back: determining XPD helicase mechanism by single‐molecule fluorescence and high‐resolution optical tweezers. DNA Repair 20:58–70. 24560558 10.1016/j.dnarep.2014.01.013 PMC4295835

[4] Conaway RC , Conaway JW (1989) An RNA polymerase II transcription factor has an associated DNA‐dependent ATPase (dATPase) activity strongly stimulated by the TATA region of promoters. Proc Natl Acad Sci USA 86:7356–7360. 2552440 10.1073/pnas.86.19.7356 PMC298060

[5] Feaver WJ , Svejstrup JQ , Bardwell L , Bardwell AJ , Buratowski S , Gulyas KD , Donahue TF , Friedberg EC , Kornberg RD (1993) Dual roles of a multiprotein complex from *S. cerevisiae* in transcription and DNA repair. Cell 75:1379–1387. 8269516 10.1016/0092-8674(93)90624-y

[6] Feaver WJ , Svejstrup JQ , Henry NL , Kornberg RD (1994) Relationship of CDK‐activating kinase and RNA polymerase II CTD kinase TFIIH/TFIIK. Cell 79:1103–1109. 8001136 10.1016/0092-8674(94)90040-x

[7] Flores O , Lu H , Reinberg D (1992) Factors involved in specific transcription by mammalian RNA polymerase II. Identification and characterization of factor IIH. J Biol Chem 267:2786–2793. 1733973

[8] Gerard M , Fischer L , Moncollin V , Chipoulet JM , Chambon P , Egly JM (1991) Purification and interaction properties of the human RNA polymerase B(II) general transcription factor BTF2. J Biol Chem 266:20940–22094. 1939143

[9] Lu H , Zawel L , Fisher L , Egly J‐M , Reinberg D (1992) Human general transcription factor IIH phosphorylates the C‐terminal domain of RNA polymerase II. Nature 358:641–645. 1495560 10.1038/358641a0

[10] Roy R , Adamczewski JP , Seroz T , Vermeulen W , Tassan JP , Schaeffer L , Nigg EA , Hoeijmakers JH , Egly JM (1994) The MO15 cell cycle kinase is associated with the TFIIH transcription‐DNA repair factor. Cell 79:1093–1101. 8001135 10.1016/0092-8674(94)90039-6

[11] Serizawa H , Makela TP , Conaway JW , Conaway RC , Weinberg RA , Young RA (1995) Association of Cdk‐activating kinase subunits with transcription factor TFIIH. Nature 374:280–282. 7885450 10.1038/374280a0

[12] Schaeffer L , Roy R , Humbert S , Moncollin V , Vermeulen W , Hoeijmakers JH , Chambon P , Egly JM (1993) DNA repair helicase: a component of BTF2 (TFIIH) basic transcription factor. Science 260:58–63. 8465201 10.1126/science.8465201

[13] Fishburn J , Tomko E , Galburt E , Hahn S (2015) Double‐stranded DNA translocase activity of transcription factor TFIIH and the mechanism of RNA polymerase II open complex formation. Proc Natl Acad Sci USA 112:3961–3966. 25775526 10.1073/pnas.1417709112 PMC4386358

[14] Grunberg S , Warfield L , Hahn S (2012) Architecture of the RNA polymerase II preinitiation complex and mechanism of ATP‐dependent promoter opening. Nat Struct Mol Biol 19:788–796. 22751016 10.1038/nsmb.2334 PMC3414687

[15] Drapkin R , Reardon JT , Ansari A , Huang JC , Zawel L , Ahn K , Sancar A , Reinberg D (1994) Dual role of TFIIH in DNA excision repair and in transcription by RNA polymerase II. Nature 368:769–772. 8152490 10.1038/368769a0

[16] Sung P , Bailly V , Weber C , Thompson LH , Prakash L , Prakash S (1993) Human xeroderma pigmentosum group D gene encodes a DNA helicase. Nature 365:852–855. 8413672 10.1038/365852a0

[17] Park E , Guzder SN , Koken MH , Jaspers‐Dekker I , Weeda G , Hoeijmakers JH , Prakash S , Prakash L (1992) RAD25 (SSL2), the yeast homolog of the human xeroderma pigmentosum group B DNA repair gene, is essential for viability. Proc Natl Acad Sci USA 89:11416–11420. 1333609 10.1073/pnas.89.23.11416 PMC50561

[18] Svejstrup JQ , Wang Z , Feave WJ , Wu X , Bushnell DA , Donahue TF , Friedberg EC , Kornberg RD (1995) Different forms of TFIIH for transcription and DNA repair: holo‐TFIIH and a nucleotide excision repairosome. Cell 80:21–28. 7813015 10.1016/0092-8674(95)90447-6

[19] Weber CA , Salazar EP , Stewart SA , Thompson LH (1990) ERCC2: cDNA cloning and molecular characterization of a human nucleotide excision repair gene with high homology to yeast RAD3. EMBO J 9:1437–1447. 2184031 10.1002/j.1460-2075.1990.tb08260.x PMC551832

[20] Di Lello P , Jenkins LM , Jones TN , Nguyen BD , Hara T , Yamaguchi H , Dikeakos JD , Appella E , Legault P , Omichinski JG (2006) Structure of the Tfb1/p53 complex: insights into the interaction between the p62/Tfb1 subunit of TFIIH and the activation domain of p53. Mol Cell 22:731–740. 16793543 10.1016/j.molcel.2006.05.007

[21] Di Lello P , Miller Jenkins LM , Mas C , Langlois C , Malitskaya E , Fradet‐Turcotte A , Archambault J , Legault P , Omichinski JG (2008) p53 and TFIIEalpha share a common binding site on the Tfb1/p62 subunit of TFIIH. Proc Natl Acad Sci USA 105:106–111. 18160537 10.1073/pnas.0707892105 PMC2224167

[22] Schilbach S , Hantsche M , Tegunov D , Dienemann C , Wigge C , Urlaub H , Cramer P (2017) Structures of transcription pre‐initiation complex with TFIIH and Mediator. Nature 551:204–209. 29088706 10.1038/nature24282 PMC6078178

[23] Fregoso M , Laine JP , Aguilar‐Fuentes J , Mocquet V , Reynaud E , Coin F , Egly JM , Zurita M (2007) DNA repair and transcriptional deficiencies caused by mutations in the Drosophila p52 subunit of TFIIH generate developmental defects and chromosome fragility. Mol Cell Biol 27:3640–3650. 17339330 10.1128/MCB.00030-07 PMC1899989

[24] Jawhari A , Laine JP , Dubaele S , Lamour V , Poterszman A , Coin F , Moras D , Egly JM (2002) p52 mediates XPB function within the transcription/repair factor TFIIH. J Biol Chem 277:31761–31667. 12080057 10.1074/jbc.M203792200

[25] Araujo SJ , Tirode F , Coin F , Pospiech H , Syvaoja JE , Stucki M , Hubscher U , Egly JM , Wood RD (2000) Nucleotide excision repair of DNA with recombinant human proteins: definition of the minimal set of factors, active forms of TFIIH, and modulation by CAK. Genes Dev 14:349–359. 10673506 PMC316364

[26] Marinoni JC , Roy R , Vermeulen W , Miniou P , Lutz Y , Weeda G , Seroz T , Gomez DM , Hoeijmakers JH , Egly JM (1997) Cloning and characterization of p52, the fifth subunit of the core of the transcription/DNA repair factor TFIIH. EMBO J 16:1093–1102. 9118947 10.1093/emboj/16.5.1093 PMC1169708

[27] Giglia‐Mari G , Coin F , Ranish JA , Hoogstraten D , Theil A , Wijgers N , Jaspers NG , Raams A , Argentini M , van der Spek PJ , Botta E , Stefanini M , Egly JM , Aebersold R , Hoeijmakers JH , Vermeulen W (2004) A new, tenth subunit of TFIIH is responsible for the DNA repair syndrome trichothiodystrophy group A. Nat Genet 36:714–719. 15220921 10.1038/ng1387

[28] Ranish JA , Hahn S , Lu Y , Yi EC , Li XJ , Eng J , Aebersold R (2004) Identification of TFB5, a new component of general transcription and DNA repair factor TFIIH. Nat Genet 36:707–713. 15220919 10.1038/ng1385

[29] Aguilar‐Fuentes J , Fregoso M , Herrera M , Reynaud E , Braun C , Egly JM , Zurita M (2008) p8/TTDA overexpression enhances UV‐irradiation resistance and suppresses TFIIH mutations in a Drosophila trichothiodystrophy model. PLoS Genet 4:e1000253. 19008953 10.1371/journal.pgen.1000253 PMC2576456

[30] Kainov DE , Vitorino M , Cavarelli J , Poterszman A , Egly JM (2008) Structural basis for group A trichothiodystrophy. Nat Struct Mol Biol 15:980–984. 19172752 10.1038/nsmb.1478

[31] Radu L , Schoenwetter E , Braun C , Marcoux J , Koelmel W , Schmitt DR , Kuper J , Cianferani S , Egly JM , Poterszman A , Kisker C (2017) The intricate network between the p34 and p44 subunits is central to the activity of the transcription/DNA repair factor TFIIH. Nucleic Acids Res 45:10872–10883. 28977422 10.1093/nar/gkx743 PMC5737387

[32] Bardwell L , Bardwell AJ , Feaver WJ , Svejstrup JQ , Kornberg RD , Friedberg EC (1994) Yeast RAD3 protein binds directly to both SSL2 and SSL1 proteins: implications for the structure and function of transcription/repair factor b. Proc Natl Acad Sci USA 91:3926–3930. 8171014 10.1073/pnas.91.9.3926 PMC43695

[33] Coin F , Marinoni JC , Rodolfo C , Fribourg S , Pedrini AM , Egly JM (1998) Mutations in the XPD helicase gene result in XP and TTD phenotypes, preventing interaction between XPD and the p44 subunit of TFIIH. Nat Genet 20:184–188. 9771713 10.1038/2491

[34] Luo J , Cimermancic P , Viswanath S , Ebmeier CC , Kim B , Dehecq M , Raman V , Greenberg CH , Pellarin R , Sali A , Taatjes DJ , Hahn S , Ranish J (2015) Architecture of the human and yeast general transcription and DNA repair factor TFIIH. Mol Cell 59:794–806. 26340423 10.1016/j.molcel.2015.07.016 PMC4560838

[35] Shiekhattar R , Mermelstein F , Fisher RP , Drapkin R , Dynlacht B , Wessling HC , Morgan DO , Reinberg D (1995) Cdk‐activating kinase complex is a component of human transcription factor TFIIH. Nature 374:283–287. 7533895 10.1038/374283a0

[36] Espinoza FH , Farrell A , Erdjument‐Bromage H , Tempst P , Morgan DO (1996) A cyclin‐dependent kinase‐activating kinase (CAK) in budding yeast unrelated to vertebrate CAK. Science 273:1714–1717. 8781234 10.1126/science.273.5282.1714

[37] Kaldis P , Sutton A , Solomon MJ (1996) The Cdk‐activating kinase (CAK) from budding yeast. Cell 86:553–564. 8752210 10.1016/s0092-8674(00)80129-4

[38] Thuret JY , Valay JG , Faye G , Mann C (1996) Civ1 (CAK in vivo), a novel Cdk‐activating kinase. Cell 86:565–576. 8752211 10.1016/s0092-8674(00)80130-0

[39] Hermand D , Pihlak A , Westerling T , Damagnez V , Vandenhaute J , Cottarel G , Makela TP (1998) Fission yeast Csk1 is a CAK‐activating kinase (CAKAK). EMBO J 17:7230–7238. 9857180 10.1093/emboj/17.24.7230 PMC1171069

[40] Lee KM , Saiz JE , Barton WA , Fisher RP (1999) Cdc2 activation in fission yeast depends on Mcs6 and Csk1, two partially redundant Cdk‐activating kinases (CAKs). Curr Biol 9:441–444. 10226032 10.1016/s0960-9822(99)80194-8

[41] Helenius K , Yang Y , Tselykh TV , Pessa HK , Frilander MJ , Makela TP (2011) Requirement of TFIIH kinase subunit Mat1 for RNA Pol II C‐terminal domain Ser5 phosphorylation, transcription and mRNA turnover. Nucleic Acids Res 39:5025–5035. 21385826 10.1093/nar/gkr107 PMC3130277

[42] Larochelle S , Batliner J , Gamble MJ , Barboza NM , Kraybill BC , Blethrow JD , Shokat KM , Fisher RP (2006) Dichotomous but stringent substrate selection by the dual‐function Cdk7 complex revealed by chemical genetics. Nat Struct Mol Biol 13:55–62. 16327805 10.1038/nsmb1028

[43] Larochelle S , Chen J , Knights R , Pandur J , Morcillo P , Erdjument‐Bromage H , Tempst P , Suter B , Fisher RP (2001) T‐loop phosphorylation stabilizes the CDK7‐cyclin H‐MAT1 complex in vivo and regulates its CTD kinase activity. EMBO J 20:3749–3759. 11447116 10.1093/emboj/20.14.3749 PMC125544

[44] Makela TP , Tassan JP , Nigg EA , Frutiger S , Hughes GJ , Weinberg RA (1994) A cyclin associated with the CDK‐activating kinase MO15. Nature 371:254–257. 8078587 10.1038/371254a0

[45] Patel SA , Simon MC (2010) Functional analysis of the Cdk7.cyclin H.Mat1 complex in mouse embryonic stem cells and embryos. J Biol Chem 285:15587–15598. 20231280 10.1074/jbc.M109.081687 PMC2865308

[46] Yankulov KY , Bentley DL (1997) Regulation of CDK7 substrate specificity by MAT1 and TFIIH. EMBO J 16:1638–1646. 9130709 10.1093/emboj/16.7.1638 PMC1169768

[47] Rossignol M , Kolb‐Cheynel I , Egly JM (1997) Substrate specificity of cdk‐activating kinase (CAK) is altered upon association with TFIIH. EMBO J 16:1628–1637. 9130708 10.1093/emboj/16.7.1628 PMC1169767

[48] Inamoto S , Segil N , Pan ZQ , Kimura M , Roeder RG (1997) The cyclin‐dependent kinase‐activating kinase (CAK) assembly factor, MAT1, targets and enhances CAK activity on the POU domains of octamer transcription factors. J Biol Chem 272:29852–29858. 9368058 10.1074/jbc.272.47.29852

[49] Ko LJ , Shieh SY , Chen X , Jayaraman L , Tamai K , Taya Y , Prives C , Pan ZQ (1997) p53 is phosphorylated by CDK7‐cyclin H in a p36MAT1‐dependent manner. Mol Cell Biol 17:7220–7229. 9372954 10.1128/mcb.17.12.7220 PMC232579

[50] Tassan JP , Jaquenoud M , Fry AM , Frutiger S , Hughes GJ , Nigg EA (1995) In vitro assembly of a functional human CDK7‐cyclin H complex requires MAT1, a novel 36 kDa RING finger protein. EMBO J 14:5608–5617. 8521818 10.1002/j.1460-2075.1995.tb00248.x PMC394676

[51] Busso D , Keriel A , Sandrock B , Poterszman A , Gileadi O , Egly JM (2000) Distinct regions of MAT1 regulate cdk7 kinase and TFIIH transcription activities. J Biol Chem 275:22815–22823. 10801852 10.1074/jbc.M002578200

[52] Fisher RP , Jin P , Chamberlin HM , Morgan DO (1995) Alternative mechanisms of CAK assembly require an assembly factor or an activating kinase. Cell 83:47–57. 7553872 10.1016/0092-8674(95)90233-3

[53] Greber BJ , Nguyen THD , Fang J , Afonine PV , Adams PD , Nogales E (2017) The cryo‐electron microscopy structure of human transcription factor IIH. Nature 549:414–417. 28902838 10.1038/nature23903 PMC5844561

[54] Gibbons BJ , Brignole EJ , Azubel M , Murakami K , Voss NR , Bushnell DA , Asturias FJ , Kornberg RD (2012) Subunit architecture of general transcription factor TFIIH. Proc Natl Acad Sci USA 109:1949–1954. 22308316 10.1073/pnas.1105266109 PMC3277522

[55] Schultz P , Fribourg S , Poterszman A , Mallouh V , Moras D , Egly JM (2000) Molecular structure of human TFIIH. Cell 102:599–607. 11007478 10.1016/s0092-8674(00)00082-9

[56] Coin F , Oksenych V , Egly JM (2007) Distinct roles for the XPB/p52 and XPD/p44 subcomplexes of TFIIH in damaged DNA opening during nucleotide excision repair. Mol Cell 26:245–256. 17466626 10.1016/j.molcel.2007.03.009

[57] Singh A , Compe E , Le May N , Egly JM (2015) TFIIH subunit alterations causing xeroderma pigmentosum and trichothiodystrophy specifically disturb several steps during transcription. Am J Hum Genet 96:194–207. 25620205 10.1016/j.ajhg.2014.12.012 PMC4320266

[58] Abdulrahman W , Iltis I , Radu L , Braun C , Maglott‐Roth A , Giraudon C , Egly JM , Poterszman A (2013) ARCH domain of XPD, an anchoring platform for CAK that conditions TFIIH DNA repair and transcription activities. Proc Natl Acad Sci USA 110:E633–E642. 23382212 10.1073/pnas.1213981110 PMC3581935

[59] He Y , Fang J , Taatjes DJ , Nogales E (2013) Structural visualization of key steps in human transcription initiation. Nature 495:481–486. 23446344 10.1038/nature11991 PMC3612373

[60] Murakami K , Tsai KL , Kalisman N , Bushnell DA , Asturias FJ , Kornberg RD (2015) Structure of an RNA polymerase II preinitiation complex. Proc Natl Acad Sci USA 112:13543–13548. 26483468 10.1073/pnas.1518255112 PMC4640751

[61] Plaschka C , Hantsche M , Dienemann C , Burzinski C , Plitzko J , Cramer P (2016) Transcription initiation complex structures elucidate DNA opening. Nature 533:353–358. 27193681 10.1038/nature17990

[62] He Y , Yan C , Fang J , Inouye C , Tjian R , Ivanov I , Nogales E (2016) Near‐atomic resolution visualization of human transcription promoter opening. Nature 533:359–365. 27193682 10.1038/nature17970 PMC4940141

[63] Allen BL , Taatjes DJ (2015) The Mediator complex: a central integrator of transcription. Nat Rev Mol Cell Biol 16:155–166. 25693131 10.1038/nrm3951 PMC4963239

[64] Dvir A , Garrett KP , Chalut C , Egly JM , Conaway JW , Conaway RC (1996) A role for ATP and TFIIH in activation of the RNA polymerase II preinitiation complex prior to transcription initiation. J Biol Chem 271:7245–7248. 8631733 10.1074/jbc.271.13.7245

[65] Goodrich JA , Tjian R (1994) Transcription factors IIE and IIH and ATP hydrolysis direct promoter clearance by RNA polymerase II. Cell 77:145–156. 8156590 10.1016/0092-8674(94)90242-9

[66] Kuper J , Braun C , Elias A , Michels G , Sauer F , Schmitt DR , Poterszman A , Egly JM , Kisker C (2014) In TFIIH, XPD helicase is exclusively devoted to DNA repair. PLoS Biol 12:e1001954. 25268380 10.1371/journal.pbio.1001954 PMC4182028

[67] Boeing S , Rigault C , Heidemann M , Eick D , Meisterernst M (2010) RNA polymerase II C‐terminal heptarepeat domain Ser‐7 phosphorylation is established in a mediator‐dependent fashion. J Biol Chem 285:188–196. 19901026 10.1074/jbc.M109.046565 PMC2804165

[68] Kim Y , Bjorklund S , Li Y , Sayre MH , Kornberg RD (1994) A multiprotein mediator of transcriptional activation and its interaction with the C‐terminal repeat domain of RNA polymerase II. Cell 77:599–608. 8187178 10.1016/0092-8674(94)90221-6

[69] Meyer KD , Lin S , Bernecky C , Gao Y , Taatjes DJ (2010) p53 activates transcription by directing structural shifts in Mediator. Nat Struct Mol Biol 17:753–760. 20453859 10.1038/nsmb.1816 PMC2932482

[70] Nair D , Kim Y , Myers LC (2005) Mediator and TFIIH govern carboxy‐terminal domain‐dependent transcription in yeast extracts. J Biol Chem 280:33739–33748. 16076843 10.1074/jbc.M506067200

[71] Esnault C , Ghavi‐Helm Y , Brun S , Soutourina J , Van Berkum N , Boschiero C , Holstege F , Werner M (2008) Mediator‐dependent recruitment of TFIIH modules in Preinitiation Complex. Mol Cell 31:337–346. 18691966 10.1016/j.molcel.2008.06.021

[72] Lee JH , Jung HS , Gunzl A (2009) Transcriptionally active TFIIH of the early‐diverged eukaryote Trypanosoma brucei harbors two novel core subunits but not a cyclin‐activating kinase complex. Nucleic Acids Res 37:3811–3820. 19386623 10.1093/nar/gkp236 PMC2699521

[73] Lee JH , Cai G , Panigrahi AK , Dunham‐Ems S , Nguyen TN , Radolf JD , Asturias FJ , Günzl A (2010) A TFIIH‐associated mediator head is a basal factor of small nuclear spliced leader RNA gene transcription in early‐diverged trypanosomes. Mol Cell Biol 30:5502–5513. 20876299 10.1128/MCB.00966-10 PMC2976424

[74] Tirode F , Busso D , Coin F , Egly JM (1999) Reconstitution of the transcription factor TFIIH: assignment of functions for the three enzymatic subunits, XPB, XPD, and cdk7. Mol Cell 3:87–95. 10024882 10.1016/s1097-2765(00)80177-x

[75] Kouzine F , Wojtowicz D , Yamane A , Resch W , Kieffer‐Kwon KR , Bandle R , Nelson S , Nakahashi H , Awasthi P , Feigenbaum L , Menoni H , Hoeijmakers J , Vermeulen W , Ge H , Przytycka TM , Levens D , Casellas R (2013) Global regulation of promoter melting in naive lymphocytes. Cell 153:988–999. 23706737 10.1016/j.cell.2013.04.033 PMC3684982

[76] Kim T , Ebright RH , Reinberg D (2000) Mechanism of ATP‐dependent promoter melting by transcription factor IIH. Science 288:1418–1421. 10827951 10.1126/science.288.5470.1418

[77] Spangler L , Wang X , Conaway JW , Conaway RC , Dvir A (2001) TFIIH action in transcription initiation and promoter escape requires distinct regions of downstream promoter DNA. Proc Natl Acad Sci USA 98:5544–5549. 11331764 10.1073/pnas.101004498 PMC33249

[78] Fishburn J , Galburt E , Hahn S (2016) Transcription start site scanning and the requirement for ATP during transcription initiation by RNA polymerase II. J Biol Chem 291:13040–13047. 27129284 10.1074/jbc.M116.724583 PMC4933221

[79] Fazal FM , Meng CA , Murakami K , Kornberg RD , Block SM (2015) Real‐time observation of the initiation of RNA polymerase II transcription. Nature 525:274–277. 26331540 10.1038/nature14882 PMC4624315

[80] Tomko EJ , Fishburn J , Hahn S , Galburt EA (2017) TFIIH generates a six‐base‐pair open complex during RNAP II transcription initiation and start‐site scanning. Nat Struct Mol Biol 24:1139–1145. 29106413 10.1038/nsmb.3500 PMC5741190

[81] Oksenych V , Bernardes de Jesus B , Zhovmer A , Egly JM , Coin F (2009) Molecular insights into the recruitment of TFIIH to sites of DNA damage. EMBO J 28:2971–2980. 19713942 10.1038/emboj.2009.230 PMC2760107

[82] Murakami K , Mattei PJ , Davis RE , Jin H , Kaplan CD , Kornberg RD (2015) Uncoupling promoter opening from start‐site scanning. Mol Cell 59:133–138. 26073544 10.1016/j.molcel.2015.05.021 PMC4490988

[83] Liu Y , Kung C , Fishburn J , Ansari AZ , Shokat KM , Hahn S (2004) Two cyclin‐dependent kinases promote RNA polymerase II transcription and formation of the scaffold complex. Mol Cell Biol 24:1721–1735. 14749387 10.1128/MCB.24.4.1721-1735.2004 PMC344185

[84] Bushnell DA , Westover KD , Davis RE , Kornberg RD (2004) Structural basis of transcription: an RNA polymerase II‐TFIIB Cocrystal at 4.5 Angstroms. Science 303:983–988. 14963322 10.1126/science.1090838

[85] Cabart P , Ujvari A , Pal M , Luse DS (2011) Transcription factor TFIIF is not required for initiation by RNA polymerase II, but it is essential to stabilize transcription factor TFIIB in early elongation complexes. Proc Natl Acad Sci USA 108:15786–15791. 21896726 10.1073/pnas.1104591108 PMC3179120

[86] Tran K , Gralla JD (2008) Control of the timing of promoter escape and RNA catalysis by the transcription factor IIb fingertip. J Biol Chem 283:15665–15671. 18411280 10.1074/jbc.M801439200 PMC2414274

[87] Moreland RJ , Tirode F , Yan Q , Conaway JW , Egly JM , Conaway RC (1999) A role for the TFIIH XPB DNA helicase in promoter escape by RNA polymerase II. J Biol Chem 274:22127–22130. 10428772 10.1074/jbc.274.32.22127

[88] Harlen KM , Churchman LS (2017) The code and beyond: transcription regulation by the RNA polymerase II carboxy‐terminal domain. Nat Rev Mol Cell Biol 18:263–273. 28248323 10.1038/nrm.2017.10

[89] Robinson PJ , Trnka MJ , Bushnell DA , Davis RE , Mattei PJ , Burlingame AL , Kornberg RD (2016) Structure of a complete mediator‐RNA polymerase II pre‐initiation complex. Cell 166:1411–1422. 27610567 10.1016/j.cell.2016.08.050 PMC5589196

[90] Naar AM , Taatjes DJ , Zhai W , Nogales E , Tjian R (2002) Human CRSP interacts with RNA polymerase II CTD and adopts a specific CTD‐bound conformation. Genes Dev 16:1339–1344. 12050112 10.1101/gad.987602 PMC186316

[91] Svejstrup JQ , Li Y , Fellows J , Gnatt A , Bjorklund S , Kornberg RD (1997) Evidence for a mediator cycle at the initiation of transcription. Proc Natl Acad Sci USA 94:6075–6078. 9177171 10.1073/pnas.94.12.6075 PMC21003

[92] Akhtar MS , Heidemann M , Tietjen JR , Zhang DW , Chapman RD , Eick D , Ansari AZ (2009) TFIIH kinase places bivalent marks on the carboxy‐terminal domain of RNA polymerase II. Mol Cell 34:387–393. 19450536 10.1016/j.molcel.2009.04.016 PMC2757088

[93] Glover‐Cutter K , Larochelle S , Erickson B , Zhang C , Shokat K , Fisher RP , Bentley DL (2009) TFIIH‐associated Cdk7 kinase functions in phosphorylation of C‐terminal domain Ser7 residues, promoter‐proximal pausing, and termination by RNA polymerase II. Mol Cell Biol 29:5455–5464. 19667075 10.1128/MCB.00637-09 PMC2756882

[94] Kim M , Suh H , Cho EJ , Buratowski S (2009) Phosphorylation of the yeast Rpb1 C‐terminal domain at serines 2, 5, and 7. J Biol Chem 284:26421–26426. 19679665 10.1074/jbc.M109.028993 PMC2785330

[95] Sogaard TM , Svejstrup JQ (2007) Hyperphosphorylation of the C‐terminal repeat domain of RNA polymerase II facilitates dissociation of its complex with mediator. J Biol Chem 282:14113–14120. 17376774 10.1074/jbc.M701345200

[96] Ebmeier CC , Erickson B , Allen BL , Allen MA , Kim H , Fong N , Jacobsen JR , Liang K , Shilatifard A , Dowell RD , Old WM , Bentley DL , Taatjes DJ (2017) Human TFIIH kinase CDK7 regulates transcription‐associated chromatin modifications. Cell Rep 20:1173–1186. 28768201 10.1016/j.celrep.2017.07.021 PMC5564226

[97] Jeronimo C , Robert F (2014) Kin28 regulates the transient association of Mediator with core promoters. Nat Struct Mol Biol 21:449–455. 24704787 10.1038/nsmb.2810 PMC3997488

[98] Wong KH , Jin Y , Struhl K (2014) TFIIH phosphorylation of the Pol II CTD stimulates mediator dissociation from the preinitiation complex and promoter escape. Mol Cell 54:601–612. 24746699 10.1016/j.molcel.2014.03.024 PMC4035452

[99] Kwak H , Lis JT (2013) Control of transcriptional elongation. Annu Rev Genet 47:483–508. 24050178 10.1146/annurev-genet-110711-155440 PMC3974797

[100] Mayer A , Lidschreiber M , Siebert M , Leike K , Soding J , Cramer P (2010) Uniform transitions of the general RNA polymerase II transcription complex. Nat Struct Mol Biol 17:1272–1278. 20818391 10.1038/nsmb.1903

[101] Booth GT , Wang IX , Cheung VG , Lis JT (2016) Divergence of a conserved elongation factor and transcription regulation in budding and fission yeast. Genome Res 26:799–811. 27197211 10.1101/gr.204578.116 PMC4889974

[102] Adelman K , Lis JT (2012) Promoter‐proximal pausing of RNA polymerase II: emerging roles in metazoans. Nat Rev Genet 13:720–731. 22986266 10.1038/nrg3293 PMC3552498

[103] Bernecky C , Plitzko JM , Cramer P (2017) Structure of a transcribing RNA polymerase II‐DSIF complex reveals a multidentate DNA‐RNA clamp. Nat Struct Mol Biol 24:809–815. 28892040 10.1038/nsmb.3465

[104] Missra A , Gilmour DS (2010) Interactions between DSIF (DRB sensitivity inducing factor), NELF (negative elongation factor), and the Drosophila RNA polymerase II transcription elongation complex. Proc Natl Acad Sci USA 107:11301–11306. 20534440 10.1073/pnas.1000681107 PMC2895096

[105] Larochelle S , Amat R , Glover‐Cutter K , Sanso M , Zhang C , Allen JJ , Shokat KM , Bentley DL , Fisher RP (2012) Cyclin‐dependent kinase control of the initiation‐to‐elongation switch of RNA polymerase II. Nat Struct Mol Biol 19:1108–1115. 23064645 10.1038/nsmb.2399 PMC3746743

[106] Nilson KA , Guo J , Turek ME , Brogie JE , Delaney E , Luse DS , Price DH (2015) THZ1 reveals roles for Cdk7 in co‐transcriptional capping and pausing. Mol Cell 59:576–587. 26257281 10.1016/j.molcel.2015.06.032 PMC4546572

[107] Diamant G , Amir‐Zilberstein L , Yamaguchi Y , Handa H , Dikstein R (2012) DSIF restricts NF‐kappaB signaling by coordinating elongation with mRNA processing of negative feedback genes. Cell Rep 2:722–731. 23041311 10.1016/j.celrep.2012.08.041

[108] Fitz J , Neumann T , Pavri R (2018) Regulation of RNA polymerase II processivity by Spt5 is restricted to a narrow window during elongation. EMBO J 37:e97965. 29514850 10.15252/embj.201797965 PMC5897773

[109] Rodriguez‐Molina JB , Tseng SC , Simonett SP , Taunton J , Ansari AZ (2016) Engineered covalent inactivation of TFIIH‐kinase reveals an elongation checkpoint and results in widespread mRNA stabilization. Mol Cell 63:433–444. 27477907 10.1016/j.molcel.2016.06.036 PMC5122673

[110] Lidschreiber M , Leike K , Cramer P (2013) Cap completion and C‐terminal repeat domain kinase recruitment underlie the initiation‐elongation transition of RNA polymerase II. Mol Cell Biol 33:3805–3816. 23878398 10.1128/MCB.00361-13 PMC3811861

[111] Shetty A , Kallgren SP , Demel C , Maier KC , Spatt D , Alver BH , Cramer P , Park PJ , Winston F (2017) Spt5 plays vital roles in the control of sense and antisense transcription elongation. Mol Cell 66:77–88. 28366642 10.1016/j.molcel.2017.02.023 PMC5394798

[112] Viladevall L , St Amour CV , Rosebrock A , Schneider S , Zhang C , Allen JJ , Shokat KM , Schwer B , Leatherwood JK , Fisher RP (2009) TFIIH and P‐TEFb coordinate transcription with capping enzyme recruitment at specific genes in fission yeast. Mol Cell 33:738–751. 19328067 10.1016/j.molcel.2009.01.029 PMC2693121

[113] Zhou Q , Li T , Price DH (2012) RNA polymerase II elongation control. Annu Rev Biochem 81:119–143. 22404626 10.1146/annurev-biochem-052610-095910 PMC4273853

[114] Sanso M , Levin RS , Lipp JJ , Wang VY , Greifenberg AK , Quezada EM , Ali A , Ghosh A , Larochelle S , Rana TM , Geyer M , Tong L , Shokat KM , Fisher RP (2016) P‐TEFb regulation of transcription termination factor Xrn2 revealed by a chemical genetic screen for Cdk9 substrates. Genes Dev 30:117–131. 26728557 10.1101/gad.269589.115 PMC4701974

[115] Ho CK , Shuman S (1999) Distinct roles for CTD Ser‐2 and Ser‐5 phosphorylation in the recruitment and allosteric activation of mammalian mRNA capping enzyme. Mol Cell 3:405–411. 10198643 10.1016/s1097-2765(00)80468-2

[116] Komarnitsky P , Cho EJ , Buratowski S (2000) Different phosphorylated forms of RNA polymerase II and associated mRNA processing factors during transcription. Genes Dev 14:2452–2460. 11018013 10.1101/gad.824700 PMC316976

[117] Schroeder SC , Schwer B , Shuman S , Bentley D (2000) Dynamic association of capping enzymes with transcribing RNA polymerase II. Genes Dev 14:2435–2440. 11018011 10.1101/gad.836300 PMC316982

[118] Schwer B , Shuman S (2011) Deciphering the RNA polymerase II CTD code in fission yeast. Mol Cell 43:311–318. 21684186 10.1016/j.molcel.2011.05.024 PMC3142328

[119] Mandal SS , Chu C , Wada T , Handa H , Shatkin AJ , Reinberg D (2004) Functional interactions of RNA‐capping enzyme with factors that positively and negatively regulate promoter escape by RNA polymerase II. Proc Natl Acad Sci USA 101:7572–7577. 15136722 10.1073/pnas.0401493101 PMC419647

[120] Pei Y , Shuman S (2002) Interactions between fission yeast mRNA capping enzymes and elongation factor Spt5. J Biol Chem 277:19639–19648. 11893740 10.1074/jbc.M200015200

[121] Chen FX , Woodfin AR , Gardini A , Rickels RA , Marshall SA , Smith ER , Shiekhattar R , Shilatifard A (2015) PAF1, a molecular regulator of promoter‐proximal pausing by RNA polymerase II. Cell 162:1003–1015. 26279188 10.1016/j.cell.2015.07.042 PMC4679144

[122] Chen FX , Xie P , Collings CK , Cao K , Aoi Y , Marshall SA , Rendleman EJ , Ugarenko M , Ozark PA , Zhang A , Shiekhattar R , Smith ER , Zhang MQ , Shilatifard A (2017) PAF1 regulation of promoter‐proximal pause release via enhancer activation. Science 357:1294–1298. 28860207 10.1126/science.aan3269 PMC6055228

[123] Yu M , Yang W , Ni T , Tang Z , Nakadai T , Zhu J , Roeder RG (2015) RNA polymerase II‐associated factor 1 regulates the release and phosphorylation of paused RNA polymerase II. Science 350:1383–1386. 26659056 10.1126/science.aad2338 PMC8729149

[124] Hong SW , Hong SM , Yoo JW , Lee YC , Kim S , Lis JT , Lee DK (2009) Phosphorylation of the RNA polymerase II C‐terminal domain by TFIIH kinase is not essential for transcription of *Saccharomyces cerevisiae* genome. Proc Natl Acad Sci USA 106:14276–14280. 19666497 10.1073/pnas.0903642106 PMC2732804

[125] Kanin EI , Kipp RT , Kung C , Slattery M , Viale A , Hahn S , Shokat KM , Ansari AZ (2007) Chemical inhibition of the TFIIH‐associated kinase cdk7/kin28 does not impair global mRNA synthesis. Proc Natl Acad Sci USA 104:5812–5817. 17392431 10.1073/pnas.0611505104 PMC1851574

[126] Moteki S , Price D (2002) Functional coupling of capping and transcription of mRNA. Mol Cell 10:599–609. 12408827 10.1016/s1097-2765(02)00660-3

[127] Suh H , Ficarro SB , Kang UB , Chun Y , Marto JA , Buratowski S (2016) Direct analysis of phosphorylation sites on the Rpb1 C‐terminal domain of RNA polymerase II. Mol Cell 61:297–304. 26799764 10.1016/j.molcel.2015.12.021 PMC4724063

[128] de la Mata M , Alonso CR , Kadener S , Fededa JP , Blaustein M , Pelisch F , Cramer P , Bentley D , Kornblihtt AR (2003) A slow RNA polymerase II affects alternative splicing in vivo. Mol Cell 12:525–532. 14536091 10.1016/j.molcel.2003.08.001

[129] Fong N , Kim H , Zhou Y , Ji X , Qiu J , Saldi T , Diener K , Jones K , Fu XD , Bentley DL (2014) Pre‐mRNA splicing is facilitated by an optimal RNA polymerase II elongation rate. Genes Dev 28:2663–2676. 25452276 10.1101/gad.252106.114 PMC4248296

[130] Munoz MJ , Perez Santangelo MS , Paronetto MP , de la Mata M , Pelisch F , Boireau S , Glover‐Cutter K , Ben‐Dov C , Blaustein M , Lozano JJ , Bird G , Bentley D , Bertrand E , Kornblihtt AR (2009) DNA damage regulates alternative splicing through inhibition of RNA polymerase II elongation. Cell 137:708–720. 19450518 10.1016/j.cell.2009.03.010

[131] Nojima T , Gomes T , Grosso AR , Kimura H , Dye MJ , Dhir S , Carmo‐Fonseca M , Proudfoot NJ (2015) Mammalian NET‐Seq reveals genome‐wide nascent transcription coupled to RNA processing. Cell 161:526–540. 25910207 10.1016/j.cell.2015.03.027 PMC4410947

[132] Mayer A , di Iulio J , Maleri S , Eser U , Vierstra J , Reynolds A , Sandstrom R , Stamatoyannopoulos JA , Churchman LS (2015) Native elongating transcript sequencing reveals human transcriptional activity at nucleotide resolution. Cell 161:541–554. 25910208 10.1016/j.cell.2015.03.010 PMC4528962

[133] Medler S , Ansari A (2015) Gene looping facilitates TFIIH kinase‐mediated termination of transcription. Sci Rep 5:12586. 26286112 10.1038/srep12586 PMC4541409

[134] Kim H , Erickson B , Luo W , Seward D , Graber JH , Pollock DD , Megee PC , Bentley DL (2010) Gene‐specific RNA polymerase II phosphorylation and the CTD code. Nat Struct Mol Biol 17:1279–1286. 20835241 10.1038/nsmb.1913 PMC3048030

[135] Wada T , Takagi T , Yamaguchi Y , Ferdous A , Imai T , Hirose S , Sugimoto S , Yano K , Hartzog GA , Winston F , Buratowski S , Handa H (1998) DSIF, a novel transcription elongation factor that regulates RNA polymerase II processivity, is composed of human Spt4 and Spt5 homologs. Genes Dev 12:343–356. 9450929 10.1101/gad.12.3.343 PMC316480

[136] Kizer KO , Phatnani HP , Shibata Y , Hall H , Greenleaf AL , Strahl BD (2005) A novel domain in Set2 mediates RNA polymerase II interaction and couples histone H3 K36 methylation with transcript elongation. Mol Cell Biol 25:3305–3316. 15798214 10.1128/MCB.25.8.3305-3316.2005 PMC1069628

[137] Lee JH , Skalnik DG (2008) Wdr82 is a C‐terminal domain‐binding protein that recruits the Setd1A Histone H3‐Lys4 methyltransferase complex to transcription start sites of transcribed human genes. Mol Cell Biol 28:609–618. 17998332 10.1128/MCB.01356-07 PMC2223426

[138] Ng HH , Robert F , Young RA , Struhl K (2003) Targeted recruitment of Set1 histone methylase by elongating Pol II provides a localized mark and memory of recent transcriptional activity. Mol Cell 11:709–719. 12667453 10.1016/s1097-2765(03)00092-3

[139] Yoh SM , Lucas JS , Jones KA (2008) The Iws1:Spt6:CTD complex controls cotranscriptional mRNA biosynthesis and HYPB/Setd2‐mediated histone H3K36 methylation. Genes Dev 22:3422–3434. 19141475 10.1101/gad.1720008 PMC2607075

[140] Deans C , Maggert KA (2015) What do you mean, “epigenetic”?. Genetics 199:887–896. 25855649 10.1534/genetics.114.173492 PMC4391566

[141] Bonasio R , Tu S , Reinberg D (2010) Molecular signals of epigenetic states. Science 330:612–616. 21030644 10.1126/science.1191078 PMC3772643

[142] Coleman RT , Struhl G (2017) Causal role for inheritance of H3K27me3 in maintaining the OFF state of a Drosophila HOX gene. Science 356:eaai8236. 28302795 10.1126/science.aai8236 PMC5595140

[143] Gaydos LJ , Wang W , Strome S (2014) Gene repression. H3K27me and PRC2 transmit a memory of repression across generations and during development. Science 345:1515–1518. 25237104 10.1126/science.1255023 PMC4238426

[144] Zenk F , Loeser E , Schiavo R , Kilpert F , Bogdanovic O , Iovino N (2017) Germ line‐inherited H3K27me3 restricts enhancer function during maternal‐to‐zygotic transition. Science 357:212–216. 28706074 10.1126/science.aam5339

[145] Morselli M , Pastor WA , Montanini B , Nee K , Ferrari R , Fu K , Bonora G , Rubbi L , Clark AT , Ottonello S , Jacobsen SE , Pellegrini M (2015) In vivo targeting of de novo DNA methylation by histone modifications in yeast and mouse. Elife 4:e06205. 25848745 10.7554/eLife.06205 PMC4412109

[146] Simon JM , Hacker KE , Singh D , Brannon AR , Parker JS , Weiser M , Ho TH , Kuan PF , Jonasch E , Furey TS , Prins JF , Lieb JD , Rathmell WK , Davis IJ (2014) Variation in chromatin accessibility in human kidney cancer links H3K36 methyltransferase loss with widespread RNA processing defects. Genome Res 24:241–250. 24158655 10.1101/gr.158253.113 PMC3912414

[147] Kolasinska‐Zwierz P , Down T , Latorre I , Liu T , Liu XS , Ahringer J (2009) Differential chromatin marking of introns and expressed exons by H3K36me3. Nat Genet 41:376–381. 19182803 10.1038/ng.322 PMC2648722

[148] Luco RF , Pan Q , Tominaga K , Blencowe BJ , Pereira‐Smith OM , Misteli T (2010) Regulation of alternative splicing by histone modifications. Science 327:996–1000. 20133523 10.1126/science.1184208 PMC2913848

[149] Sims RJ, 3rd , Millhouse S , Chen CF , Lewis BA , Erdjument‐Bromage H , Tempst P , Manley JL , Reinberg D (2007) Recognition of trimethylated histone H3 lysine 4 facilitates the recruitment of transcription postinitiation factors and pre‐mRNA splicing. Mol Cell 28:665–676. 18042460 10.1016/j.molcel.2007.11.010 PMC2276655

[150] Lu H , Fisher RP , Bailey P , Levine AJ (1997) The CDK7‐cycH‐p36 complex of transcription factor IIH phosphorylates p53, enhancing its sequence‐specific DNA binding activity in vitro. Mol Cell Biol 17:5923–5934. 9315650 10.1128/mcb.17.10.5923 PMC232440

[151] Chen D , Riedl T , Washbrook E , Pace PE , Coombes RC , Egly JM , Ali S (2000) Activation of estrogen receptor alpha by S118 phosphorylation involves a ligand‐dependent interaction with TFIIH and participation of CDK7. Mol Cell 6:127–137. 10949034

[152] Chymkowitch P , Le May N , Charneau P , Compe E , Egly JM (2011) The phosphorylation of the androgen receptor by TFIIH directs the ubiquitin/proteasome process. embo J 30:468–479. 21157430 10.1038/emboj.2010.337 PMC3034013

[153] Keriel A , Stary A , Sarasin A , Rochette‐Egly C , Egly JM (2002) XPD mutations prevent TFIIH‐dependent transactivation by nuclear receptors and phosphorylation of RARalpha. Cell 109:125–135. 11955452 10.1016/s0092-8674(02)00692-x

[154] Rochette‐Egly C , Adam S , Rossignol M , Egly JM , Chambon P (1997) Stimulation of RAR alpha activation function AF‐1 through binding to the general transcription factor TFIIH and phosphorylation by CDK7. Cell 90:97–107. 9230306 10.1016/s0092-8674(00)80317-7

[155] Beltrao P , Albanese V , Kenner LR , Swaney DL , Burlingame A , Villen J , Lim WA , Fraser JS , Frydman J , Krogan NJ (2012) Systematic functional prioritization of protein posttranslational modifications. Cell 150:413–425. 22817900 10.1016/j.cell.2012.05.036 PMC3404735

[156] Holstege FCP , van der Vliet PC , Timmers HTM (1996) Opening of an RNA polymerase II promoter occurs in two distinct steps and requires the basal transcription factors IIE and IIH. EMBO J 14:810–819. PMC450078 8612591

[157] Ohkuma Y , Roeder RG (1994) Regulation of TFIIH ATPase and kinase activities by TFIIE during active initiation complex formation. Nature 368:160–163. 8166891 10.1038/368160a0

[158] Plaschka C , Lariviere L , Wenzeck L , Seizl M , Hemann M , Tegunov D , Petrotchenko EV , Borchers CH , Baumeister W , Herzog F , Villa E , Cramer P (2015) Architecture of the RNA polymerase II‐mediator core initiation complex. Nature 518:376–380. 25652824 10.1038/nature14229

[159] Akoulitchev S , Chuikov S , Reinberg D (2000) TFIIH is negatively regulated by cdk8‐containing mediator complexes. Nature 407:102–106. 10993082 10.1038/35024111

[160] Malik S , Molina H , Xue Z (2017) PIC activation through functional interplay between mediator and TFIIH. J Mol Biol 429:48–63. 27916598 10.1016/j.jmb.2016.11.026 PMC5186404

[161] Winkler GS , Araujo SJ , Fiedler U , Vermeulen W , Coin F , Egly JM , Hoeijmakers JH , Wood RD , Timmers HT , Weeda G (2000) TFIIH with inactive XPD helicase functions in transcription initiation but is defective in DNA repair. J Biol Chem 275:4258–4266. 10660593 10.1074/jbc.275.6.4258

[162] Yoder K , Sarasin A , Kraemer K , McIlhatton M , Bushman F , Fishel R (2006) The DNA repair genes XPB and XPD defend cells from retroviral infection. Proc Natl Acad Sci USA 103:4622–4627. 16537383 10.1073/pnas.0509828103 PMC1450221

[163] Yoder KE , Roddick W , Hoellerbauer P , Fishel R (2011) XPB mediated retroviral cDNA degradation coincides with entry to the nucleus. Virology 410:291–298. 21167544 10.1016/j.virol.2010.11.016 PMC3030651

[164] Gray LT , Vallur AC , Eddy J , Maizels N (2014) G quadruplexes are genomewide targets of transcriptional helicases XPB and XPD. Nat Chem Biol 10:313–318. 24609361 10.1038/nchembio.1475 PMC4006364

[165] Rhodes D , Lipps HJ (2015) G‐quadruplexes and their regulatory roles in biology. Nucleic Acids Res 43:8627–8637. 26350216 10.1093/nar/gkv862 PMC4605312

[166] Rossi DJ , Londesborough A , Korsisaari N , Pihlak A , Lehtonen E , Henkemeyer M , Makela TP (2001) Inability to enter S phase and defective RNA polymerase II CTD phosphorylation in mice lacking Mat1. EMBO J 20:2844–2856. 11387217 10.1093/emboj/20.11.2844 PMC125252

[167] Larochelle S , Merrick KA , Terret ME , Wohlbold L , Barboza NM , Zhang C , Shokat KM , Jallepalli PV , Fisher RP (2007) Requirements for Cdk7 in the assembly of Cdk1/cyclin B and activation of Cdk2 revealed by chemical genetics in human cells. Mol Cell 25:839–850. 17386261 10.1016/j.molcel.2007.02.003 PMC1858677

[168] Fisher RP (2005) Secrets of a double agent: CDK7 in cell‐cycle control and transcription. J Cell Sci 118:5171–5180. 16280550 10.1242/jcs.02718

[169] Merrick KA , Larochelle S , Zhang C , Allen JJ , Shokat KM , Fisher RP (2008) Distinct activation pathways confer cyclin‐binding specificity on Cdk1 and Cdk2 in human cells. Mol Cell 32:662–672. 19061641 10.1016/j.molcel.2008.10.022 PMC2643088

[170] Schachter MM , Merrick KA , Larochelle S , Hirschi A , Zhang C , Shokat KM , Rubin SM , Fisher RP (2013) A Cdk7‐Cdk4 T‐loop phosphorylation cascade promotes G1 progression. Mol Cell 50:250–260. 23622515 10.1016/j.molcel.2013.04.003 PMC3677717

[171] Larochelle S , Pandur J , Fisher RP , Salz HK , Suter B (1998) Cdk7 is essential for mitosis and for in vivo Cdk‐activating kinase activity. Genes Dev 12:370–381. 9450931 10.1101/gad.12.3.370 PMC316490

[172] Abdullah AI , Zhang H , Nie Y , Tang W , Sun T (2016) CDK7 and miR‐210 co‐regulate cell‐cycle progression of neural progenitors in the developing neocortex. Stem Cell Rep 7:69–79. 10.1016/j.stemcr.2016.06.005 PMC4944761 27411104

[173] Ganuza M , Sáiz‐Ladera C , Cañamero M , Gómez G , Schneider R , Blasco MA , Pisano D , Paramio JM , Santamaría D , Barbacid M (2012) Genetic inactivation of Cdk7 leads to cell cycle arrest and induces premature aging due to adult stem cell exhaustion. EMBO J 31:2498–2510. 22505032 10.1038/emboj.2012.94 PMC3365431

[174] Luo S , Horvitz HR (2017) The CDK8 complex and proneural proteins together drive neurogenesis from a mesodermal lineage. Curr Biol 27:661–672. 28238659 10.1016/j.cub.2017.01.056 PMC5384724

[175] He G , Yang X , Wang G , Qi J , Mao R , Wu Z , Zhou Z (2017) Cdk7 is required for activity‐dependent neuronal gene expression, long‐lasting synaptic plasticity and long‐term memory. Front Mol Neurosci 10:365. 29163040 10.3389/fnmol.2017.00365 PMC5681959

[176] Korzus E , Rosenfeld MG , Mayford M (2004) CBP histone acetyltransferase activity is a critical component of memory consolidation. Neuron 42:961–972. 15207240 10.1016/j.neuron.2004.06.002 PMC8048715

[177] Cleaver JE , Lam ET , Revet I (2009) Disorders of nucleotide excision repair: the genetic and molecular basis of heterogeneity. Nat Rev Genet 10:756–768. 19809470 10.1038/nrg2663

[178] Dubaele S , Proietti De Santis L , Bienstock RJ , Keriel A , Stefanini M , Van Houten B , Egly JM (2003) Basal transcription defect discriminates between xeroderma pigmentosum and trichothiodystrophy in XPD patients. Mol Cell 11:1635–1646. 12820975 10.1016/s1097-2765(03)00182-5

[179] Moriel‐Carretero M , Herrera‐Moyano E , Aguilera A (2015) A unified model for the molecular basis of xeroderma pigmentosum‐Cockayne syndrome. Rare Dis 3:e1079362. 26460500 10.1080/21675511.2015.1079362 PMC4588225

[180] Mourgues S , Gautier V , Lagarou A , Bordier C , Mourcet A , Slingerland J , Kaddoum L , Coin F , Vermeulen W , Gonzales de Peredo A , Monsarrat B , Mari PO , Giglia‐Mari G (2013) ELL, a novel TFIIH partner, is involved in transcription restart after DNA repair. Proc Natl Acad Sci USA 110:17927–17932. 24127601 10.1073/pnas.1305009110 PMC3816466

[181] Velez‐Cruz R , Zadorin AS , Coin F , Egly JM (2013) Sirt1 suppresses RNA synthesis after UV irradiation in combined xeroderma pigmentosum group D/Cockayne syndrome (XP‐D/CS) cells. Proc Natl Acad Sci USA 110:E212–E220. 23267107 10.1073/pnas.1213076110 PMC3549127

[182] Guzder SN , Sung P , Prakash S , Prakash L (1995) Lethality in yeast of trichothiodystrophy (TTD) mutations in the human xeroderma pigmentosum group D gene. Implications for transcriptional defect in TTD. J Biol Chem 270:17660–17663. 7629061 10.1074/jbc.270.30.17660

[183] Hashimoto S , Egly JM (2009) Trichothiodystrophy view from the molecular basis of DNA repair/transcription factor TFIIH. Hum Mol Genet 18:R224–R230. 19808800 10.1093/hmg/ddp390

[184] Jaitovich‐Groisman I , Benlimame N , Slagle BL , Perez MH , Alpert L , Song DJ , Fotouhi‐Ardakani N , Galipeau J , Alaoui‐Jamali MA (2001) Transcriptional regulation of the TFIIH transcription repair components XPB and XPD by the hepatitis B virus x protein in liver cells and transgenic liver tissue. J Biol Chem 276:14124–14132. 11278765 10.1074/jbc.M010852200

[185] Le May N , Dubaele S , Proietti De Santis L , Billecocq A , Bouloy M , Egly JM (2004) TFIIH transcription factor, a target for the Rift Valley hemorrhagic fever virus. Cell 116:541–550. 14980221 10.1016/s0092-8674(04)00132-1

[186] Cyr N , de la Fuente C , Lecoq L , Guendel I , Chabot PR , Kehn‐Hall K , Omichinski JG (2015) A OmegaXaV motif in the Rift Valley fever virus NSs protein is essential for degrading p62, forming nuclear filaments and virulence. Proc Natl Acad Sci USA 112:6021–6026. 25918396 10.1073/pnas.1503688112 PMC4434773

[187] Kalveram B , Lihoradova O , Ikegami T (2011) NSs protein of rift valley fever virus promotes posttranslational downregulation of the TFIIH subunit p62. J Virol 85:6234–6243. 21543505 10.1128/JVI.02255-10 PMC3126510

[188] Cujec TP , Okamoto H , Fujinaga K , Meyer J , Chamberlin H , Morgan DO , Peterlin BM (1997) The HIV transactivator TAT binds to the CDK‐activating kinase and activates the phosphorylation of the carboxy‐terminal domain of RNA polymerase II. Genes Dev 11:2645–2657. 9334327 10.1101/gad.11.20.2645 PMC316603

[189] Parada CA , Roeder RG (1996) Enhanced processivity of RNA polymerase II triggered by Tat‐induced phosphorylation of its carboxy‐terminal domain. Nature 384:375–378. 8934526 10.1038/384375a0

[190] Kim YK , Bourgeois CF , Pearson R , Tyagi M , West MJ , Wong J , Wu SY , Chiang CM , Karn J (2006) Recruitment of TFIIH to the HIV LTR is a rate‐limiting step in the emergence of HIV from latency. EMBO J 25:3596–3604. 16874302 10.1038/sj.emboj.7601248 PMC1538560

[191] Hutterer C , Eickhoff J , Milbradt J , Korn K , Zeittrager I , Bahsi H , Wagner S , Zischinsky G , Wolf A , Degenhart C , Unger A , Baumann M , Klebl B , Marschall M (2015) A novel CDK7 inhibitor of the Pyrazolotriazine class exerts broad‐spectrum antiviral activity at nanomolar concentrations. Antimicrob Agents Chemother 59:2062–2071. 25624324 10.1128/AAC.04534-14 PMC4356785

[192] Li B , Ni Chonghaile T , Fan Y , Madden SF , Klinger R , O'Connor AE , Walsh L , O'Hurley G , Mallya Udupi G , Joseph J , Tarrant F , Conroy E , Gaber A , Chin SF , Bardwell HA , Provenzano E , Crown J , Dubois T , Linn S , Jirstrom K , Caldas C , O'Connor DP , Gallagher WM (2017) Therapeutic rationale to target highly expressed CDK7 conferring poor outcomes in triple‐negative breast cancer. Cancer Res 77:3834–3845. 28455421 10.1158/0008-5472.CAN-16-2546

[193] Patel H , Abduljabbar R , Lai C‐F , Periyasamy M , Harrod A , Gemma C , Steel JH , Patel N , Busonero C , Jerjees D , Remenyi J , Smith S , Gomm JJ , Magnani L , Győrffy B , Jones LJ , Fuller‐Pace F , Shousha S , Buluwela L , Rakha EA , Ellis IO , Coombes RC , Ali S (2016) Expression of CDK7, cyclin H, and MAT1 is elevated in breast cancer and is prognostic in estrogen receptor‐positive breast cancer. Clin Cancer Res 22:5929–5938. 27301701 10.1158/1078-0432.CCR-15-1104 PMC5293170

[194] Hnisz D , Abraham BJ , Lee TI , Lau A , Saint‐Andre V , Sigova AA , Hoke HA , Young RA (2013) Super‐enhancers in the control of cell identity and disease. Cell 155:934–947. 24119843 10.1016/j.cell.2013.09.053 PMC3841062

[195] Loven J , Hoke HA , Lin CY , Lau A , Orlando DA , Vakoc CR , Bradner JE , Lee TI , Young RA (2013) Selective inhibition of tumor oncogenes by disruption of super‐enhancers. Cell 153:320–334. 23582323 10.1016/j.cell.2013.03.036 PMC3760967

[196] Pott S , Lieb JD (2015) What are super‐enhancers?. Nat Genet 47:8–12. 25547603 10.1038/ng.3167

[197] Hnisz D , Shrinivas K , Young RA , Chakraborty AK , Sharp PA (2017) A phase separation model for transcriptional control. Cell 169:13–23. 28340338 10.1016/j.cell.2017.02.007 PMC5432200

[198] Chipumuro E , Marco E , Christensen CL , Kwiatkowski N , Zhang T , Hatheway CM , Abraham BJ , Sharma B , Yeung C , Altabef A , Perez‐Atayde A , Wong KK , Yuan GC , Gray NS , Young RA , George RE (2014) CDK7 inhibition suppresses super‐enhancer‐linked oncogenic transcription in MYCN‐driven cancer. Cell 159:1126–1139. 25416950 10.1016/j.cell.2014.10.024 PMC4243043

[199] Christensen CL , Kwiatkowski N , Abraham BJ , Carretero J , Al‐Shahrour F , Zhang T , Chipumuro E , Herter‐Sprie GS , Akbay EA , Altabef A , Zhang J , Shimamura T , Capelletti M , Reibel JB , Cavanaugh JD , Gao P , Liu Y , Michaelsen SR , Poulsen HS , Aref AR , Barbie DA , Bradner JE , George RE , Gray NS , Young RA , Wong KK (2014) Targeting transcriptional addictions in small cell lung cancer with a covalent CDK7 inhibitor. Cancer Cell 26:909–922. 25490451 10.1016/j.ccell.2014.10.019 PMC4261156

[200] Kwiatkowski N , Zhang T , Rahl PB , Abraham BJ , Reddy J , Ficarro SB , Dastur A , Amzallag A , Ramaswamy S , Tesar B , Jenkins CE , Hannett NM , McMillin D , Sanda T , Sim T , Kim ND , Look T , Mitsiades CS , Weng AP , Brown JR , Benes CH , Marto JA , Young RA , Gray NS (2014) Targeting transcription regulation in cancer with a covalent CDK7 inhibitor. Nature 511:616–620. 25043025 10.1038/nature13393 PMC4244910

[201] Wang Y , Zhang T , Kwiatkowski N , Abraham BJ , Lee TI , Xie S , Yuzugullu H , Von T , Li H , Lin Z , Stover DG , Lim E , Wang ZC , Iglehart JD , Young RA , Gray NS , Zhao JJ (2015) CDK7‐dependent transcriptional addiction in triple‐negative breast cancer. Cell 163:174–186. 26406377 10.1016/j.cell.2015.08.063 PMC4583659

[202] Cayrol F , Praditsuktavorn P , Fernando TM , Kwiatkowski N , Marullo R , Calvo‐Vidal MN , Phillip J , Pera B , Yang SN , Takpradit K , Roman L , Gaudiano M , Crescenzo R , Ruan J , Inghirami G , Zhang T , Cremaschi G , Gray NS , Cerchietti L (2017) THZ1 targeting CDK7 suppresses STAT transcriptional activity and sensitizes T‐cell lymphomas to BCL2 inhibitors. Nat Commun 8:14290. 28134252 10.1038/ncomms14290 PMC5290269

[203] Kalan S , Amat R , Schachter MM , Kwiatkowski N , Abraham BJ , Liang Y , Zhang T , Olson CM , Larochelle S , Young RA , Gray NS , Fisher RP (2017) Activation of the p53 transcriptional program sensitizes cancer cells to Cdk7 inhibitors. Cell Rep 21:467–481. 29020632 10.1016/j.celrep.2017.09.056 PMC5687273

[204] Rusan M , Li K , Li Y , Christensen CL , Abraham BJ , Kwiatkowski N , Buczkowski KA , Bockorny B , Chen T , Li S , Rhee K , Zhang H , Chen W , Terai H , Tavares T , Leggett AL , Li T , Wang Y , Zhang T , Kim TJ , Hong SH , Poudel‐Neupane N , Silkes M , Mudianto T , Tan L , Shimamura T , Meyerson M , Bass AJ , Watanabe H , Gray NS , Young RA , Wong KK , Hammerman PS (2018) Suppression of adaptive responses to targeted cancer therapy by transcriptional repression. Cancer Discov 8:59–73. 29054992 10.1158/2159-8290.CD-17-0461 PMC5819998

[205] Kelso TW , Baumgart K , Eickhoff J , Albert T , Antrecht C , Lemcke S , Klebl B , Meisterernst M (2014) Cyclin‐dependent kinase 7 controls mRNA synthesis by affecting stability of preinitiation complexes, leading to altered gene expression, cell cycle progression, and survival of tumor cells. Mol Cell Biol 34:3675–3688. 25047832 10.1128/MCB.00595-14 PMC4187722

[206] Bishop AC , Ubersax JA , Petsch DT , Matheos DP , Gray NS , Blethrow J , Shimizu E , Tsien JZ , Schultz PG , Rose MD , Wood JL , Morgan DO , Shokat KM (2000) A chemical switch for inhibitor‐sensitive alleles of any protein kinase. Nature 407:395–401. 11014197 10.1038/35030148

[207] He QL , Titov DV , Li J , Tan M , Ye Z , Zhao Y , Romo D , Liu JO (2015) Covalent modification of a cysteine residue in the XPB subunit of the general transcription factor TFIIH through single epoxide cleavage of the transcription inhibitor triptolide. Angew Chem Int Ed Engl 54:1859–1863. 25504624 10.1002/anie.201408817 PMC4314353

[208] Titov DV , Gilman B , He QL , Bhat S , Low WK , Dang Y , Smeaton M , Demain AL , Miller PS , Kugel JF , Goodrich JA , Liu JO (2011) XPB, a subunit of TFIIH, is a target of the natural product triptolide. Nat Chem Biol 7:182–188. 21278739 10.1038/nchembio.522 PMC3622543

[209] Chen F , Gao X , Shilatifard A (2015) Stably paused genes revealed through inhibition of transcription initiation by the TFIIH inhibitor triptolide. Genes Dev 29:39–47. 25561494 10.1101/gad.246173.114 PMC4281563

[210] Manzo SG , Zhou ZL , Wang YQ , Marinello J , He JX , Li YC , Ding J , Capranico G , Miao ZH (2012) Natural product triptolide mediates cancer cell death by triggering CDK7‐dependent degradation of RNA polymerase II. Cancer Res 72:5363–5373. 22926559 10.1158/0008-5472.CAN-12-1006

[211] Wang Y , Lu JJ , He L , Yu Q (2011) Triptolide (TPL) inhibits global transcription by inducing proteasome‐dependent degradation of RNA polymerase II (Pol II). PLoS One 6:e23993. 21931633 10.1371/journal.pone.0023993 PMC3172214

[212] Jonkers I , Kwak H , Lis JT (2014) Genome‐wide dynamics of Pol II elongation and its interplay with promoter proximal pausing, chromatin, and exons. Elife 3:e02407. 24843027 10.7554/eLife.02407 PMC4001325

[213] Core LJ , Waterfall JJ , Lis JT (2008) Nascent RNA sequencing reveals widespread pausing and divergent initiation at human promoters. Science 322:1845–1848. 19056941 10.1126/science.1162228 PMC2833333

[214] Alekseev S , Ayadi M , Brino L , Egly JM , Larsen AK , Coin F (2014) A small molecule screen identifies an inhibitor of DNA repair inducing the degradation of TFIIH and the chemosensitization of tumor cells to platinum. Chem Biol 21:398–407. 24508195 10.1016/j.chembiol.2013.12.014

[215] Alekseev S , Nagy Z , Sandoz J , Weiss A , Egly JM , Le May N , Coin F (2017) Transcription without XPB establishes a unified helicase‐independent mechanism of promoter opening in eukaryotic gene expression. Mol Cell 65:504–514. 28157507 10.1016/j.molcel.2017.01.012

[216] Tee WW , Shen SS , Oksuz O , Narendra V , Reinberg D (2014) Erk1/2 activity promotes chromatin features and RNAPII phosphorylation at developmental promoters in mouse ESCs. Cell 156:678–690. 24529373 10.1016/j.cell.2014.01.009 PMC4006806

[217] Baell J , Walters MA (2014) Chemistry: Chemical con artists foil drug discovery. Nature 513:481–483. 25254460 10.1038/513481a

[218] Warfield L , Luo J , Ranish J , Hahn S (2016) Function of conserved topological regions within the *Saccharomyces cerevisiae* basal transcription factor TFIIH. Mol Cell Biol 36:2464–2475. 27381459 10.1128/MCB.00182-16 PMC5021372

[219] Murakami K , Gibbons BJ , Davis RE , Nagai S , Liu X , Robinson PJ , Wu T , Kaplan CD , Kornberg RD (2012) Tfb6, a previously unidentified subunit of the general transcription factor TFIIH, facilitates dissociation of Ssl2 helicase after transcription initiation. Proc Natl Acad Sci USA 109:4816–4821. 22411836 10.1073/pnas.1201448109 PMC3323989

[220] Louder RK , He Y , López‐Blanco JR , Fang J , Chacón P , Nogales E (2016) Structure of promoter‐bound TFIID and model of human pre‐initiation complex assembly. Nature 531:604–609. 27007846 10.1038/nature17394 PMC4856295

[221] Gegonne A , Weissman JD , Lu H , Zhou M , Dasgupta A , Ribble R , Brady JN , Singer DS (2008) TFIID component TAF7 functionally interacts with both TFIIH and P‐TEFb. Proc Natl Acad Sci USA 105:5367–5372. 18391197 10.1073/pnas.0801637105 PMC2291086

[222] Yudkovsky N , Ranish JA , Hahn S (2000) A transcription reinitiation intermediate that is stabilized by activator. Nature 408:225–229. 11089979 10.1038/35041603

[223] Seila AC , Calabrese JM , Levine SS , Yeo GW , Rahl PB , Flynn RA , Young RA , Sharp PA (2008) Divergent transcription from active promoters. Science 322:1849–1851. 19056940 10.1126/science.1162253 PMC2692996

[224] Okuda M , Kinoshita M , Kakumu E , Sugasawa K , Nishimura Y (2015) Structural insight into the mechanism of TFIIH recognition by the acidic string of the nucleotide excision repair factor XPC. Structure 23:1827–1837. 26278177 10.1016/j.str.2015.07.009

[225] Okuda M , Nakazawa Y , Guo C , Ogi T , Nishimura Y (2017) Common TFIIH recruitment mechanism in global genome and transcription‐coupled repair subpathways. Nucleic Acids Res 45:13043–13055. 29069470 10.1093/nar/gkx970 PMC5727438

[226] Ruthemann P , Balbo Pogliano C , Codilupi T , Garajova Z , Naegeli H (2017) Chromatin remodeler CHD1 promotes XPC‐to‐TFIIH handover of nucleosomal UV lesions in nucleotide excision repair. EMBO J 36:3372–3386. 29018037 10.15252/embj.201695742 PMC5686551

[227] Damodaren N , Van Eeuwen T , Zamel J , Lin‐Shiao E , Kalisman N , Murakami K (2017) Def1 interacts with TFIIH and modulates RNA polymerase II transcription. Proc Natl Acad Sci USA 114:13230–13235. 29180430 10.1073/pnas.1707955114 PMC5740667

